# Effects of Single and Repeated Oral Doses of Ochratoxin A on the Lipid Peroxidation and Antioxidant Defense Systems in Mouse Kidneys

**DOI:** 10.3390/toxins12110732

**Published:** 2020-11-22

**Authors:** Szilamér Ferenczi, Dániel Kuti, Mátyás Cserháti, Csilla Krifaton, Sándor Szoboszlay, József Kukolya, Zsuzsanna Szőke, Mihály Albert, Balázs Kriszt, Krisztina J. Kovács, Miklós Mézes, Krisztián Balogh

**Affiliations:** 1 Institute of Experimental Medicine, Laboratory of Molecular Neuroendocrinology, 43. Szigony Street, Budapest 1083, Hungary; kuti.daniel@koki.mta.hu (D.K.); kovacs.krisztina@koki.mta.hu (K.J.K.); 2 Szent István University, Department of Environmental Protection & Safety, 1. Páter K. Street, Gödöllő 2100, Hungary; Cserhati.Matyas@mkk.szie.hu (M.C.); Krifaton.Csilla@mkk.szie.hu (C.K.); szoboszlay.sandor@mkk.szie.hu (S.S.); Kriszt.Balazs@mkk.szie.hu (B.K.); 3 Central Environmental and Food Science Research Institute, Department of Microbiology, 15. Herman O. Street, Budapest 1022, Hungary; kukolya.jozsef@gmail.com; 4 National Agricultural Research and Innovation Center, Agricultural Biotechnology Institute, Reproduction Biology and Toxicology Research Group, 4. Szent-Györgyi A. Street, Gödöllő 2100, Hungary; szoke.zsuzsanna@abc.naik.hu; 5 CEVA Phylaxia Ltd, 5. Szállás Street, Budapest 1107, Hungary; mihaly.albert@ceva.com; 6 Szent István University, Department of Nutrition, 1. Páter K. Street, Gödöllő 2100, Hungary; Mezes.Miklos@mkk.szie.hu (M.M.); Balogh.Krisztian@mkk.szie.hu (K.B.); 7 “MTA-KE-SZIE Mycotoxins in the Food Chain” Research Group, Hungarian Academy of Science, Kaposvár University, 40. Guba Sándor Street, Kaposvár 7400, Hungary

**Keywords:** ochratoxin-A, glutathione, oxidative stress, kidney, gene expression

## Abstract

Ochratoxin-A (OTA) is a carcinogenic and nephrotoxic mycotoxin, which may cause health problems in humans and animals, and it is a contaminant in foods and feeds. The purpose of the present study is to evaluate the effect of oral OTA exposure on the antioxidant defense and lipid peroxidation in the kidney. In vivo administration of OTA in CD1, male mice (1 or 10 mg/kg body weight in a single oral dose for 24 h and repeated daily oral dose for 72 h or repeated daily oral dose of 0.5 mg/kg bodyweight for 21 days) resulted in a significant elevation of OTA levels in blood plasma. Some histopathological alterations, transcriptional changes in the glutathione system, and oxidative stress response-related genes were also found. In the renal cortex, the activity of the glutathione-system-related enzymes and certain metabolites of the lipid peroxidation (conjugated dienes, trienes, and thiobarbituric reactive substances) also changed.

## 1. Introduction

Mycotoxins are harmful or toxic to animals and humans. Ochratoxin-A (OTA) is a secondary metabolite of certain *Aspergillus* and *Penicillium* strains [1]. OTA exposure caused nephropathy in porcine [2,3] and humans [4,5,6,7]. OTA-induced nephropathy is characterized by cell degeneration of renal proximal tubular and glomerular epithelial cells in the renal cortex area. It can also cause interstitial fibrosis, polyuria, and alterations in hematological and clinical biochemical parameters [8]. The chemical structure of OTA is similar to phenylalanine; thus, it impairs protein synthesis [9,10]. As a carcinogen, OTA has been demonstrated in renal adenocarcinoma and liver tumors [11,12]. OTA affects the expression of genes related to cell damage, apoptosis, cellular stress, and antioxidant defense [13]. OTA toxicity mechanism includes the formation of oxygen free radicals and lipid peroxidation [14]. There is evidence that antioxidants, such as vitamins E and C, decrease lipid peroxide formation, therefore, the oxidative stress-inducing effect of OTA [15]. However, renal tubular cell damage can be found without the induction of lipid peroxidation but a higher expression of genes encoding the antioxidant enzymes [16]. The molecular mechanisms that respond to oxidative stress are highly conserved in mammals. The transcription factor nuclear factor erythroid 2-related factor 2 (NRF2) is the master regulator of the oxidative stress response [17]. OTA-induced oxidative stress affects the antioxidant defense systems [18] and also modulates NRF2 expression and, therefore, activation of the antioxidant response element [19]. In response to elevated ROS levels, NRF2 induces the expression of certain antioxidant enzymes such as Heme Oxygenase (HO1), NAD(P)H quinine dehydrogenase (NQO1), and glutathione synthetase (GSS) [20].

These factors belong to the phase II detoxifying enzymes in xenobiotic transformation [21]. The activity of the NRF2 is regulated by redox balance in the cells. In physiological conditions, NRF2 is bound to Ketch-like ECH-associated protein 1 (KEAP1) as an oxidative stress sensor and promote ubiquitination and enzymatic degradation of NRF2 in proteasomes [22], keeping the low basal NRF2 activity. However, when oxidative stress increases, the KEAP1 cysteine side chains are oxidized. Consequently, the interaction between NRF2 and KEAP1 destabilizes, allowing the nuclear translocation of NRF2 to transcribe its specific target genes [23]. The principal element of the antioxidant system is the superoxide dismutase (SOD) enzyme family. SOD1 is the intracellular and nuclear form of SOD, accounting for 80% of total SOD protein [24]. SOD2 is localized in the mitochondria [24], and SOD3 is the secreted form that is associated with the extracellular matrix [25]. The SOD enzymes are scavenged reactive oxygen species, mainly superoxide anion generated in the cell by NADPH oxidase (NOX), xanthine oxidase, cytochrome P450, and mitochondrial respiration [26]. The SOD1 is a copper/zinc-dependent enzyme, and zinc supplementation could decrease OTA-induced oxidative damage in HepG2 cells [27]. The in vivo effect of acute and chronic OTA exposition on the kidney SOD enzymes is poorly understood. A recently identified component of the antioxidant defense is the HACE1-HECT E3 ligase. HACE1 is a tumor suppressor that ubiquitylates and proteasomal degradation of RAC1 protein (GTP-bound form of the Rho family GTPase). This event inhibits the de novo ROS generation by RAC1-dependent NADPH oxidases. It will thereby confer the cellular defense from oxidative DNA damage and hyper-proliferation [28]. Inactivation of RAC1 reduced NADPH oxidase I-dependent ROS production [29]. The roles of HACE1 and RAC1 in the OTA toxicities have not been investigated previously. 

The effects of OTA on molecular redox mechanisms have been reported; however, the sequence of induction remains unsolved. Further, in vivo is essential to understand the toxic efficiency mode of action of OTA on the renal cortical area, which is the most OTA-affected tissue organ in animals and humans. The present study aimed to investigate the toxic effect of OTA-related oxidative stress and lipid peroxidation in the renal cortex in mice. The use of a rodent based in vivo toxicological study is the most suitable approach. The following markers were analyzed: alterations in the kidney and spleen weight, changes in the expression of OTA-affected genes, and activities of their products in the kidney [13]. In addition, lipid peroxidation parameters, activities of antioxidant enzymes, and immunochemical techniques (Western blot and ELISA) were also included in this study.

## 2. Results

### 2.1. Water and Food Consumption and Body Weight Change in Mice

Consumption of food and water did not change as an effect of the single oral dose (24 h), repeated daily oral dose (72 h), or repeated daily oral dose (21 days) OTA treatment. The bodyweight of the treated animals in repeated daily oral dose (72 h) and repeated daily oral dose (21 days) experiment also did not alter significantly (data not shown).

### 2.2. Blood Plasma OTA Content in a Single Oral Dose (24 h), Repeated Daily Oral Dose (72 h), and Repeated Daily Oral Dose (21 Days) Treatments 

Low levels of OTA were found in the blood plasma of vehicle or methyl-methanesulfonate (MMS)-treated control animals. Single oral dose (24 h) mycotoxin administration significantly increased the OTA concentration in the blood plasma in 1 mg/kg bw and 10 mg/kg bw dose groups (Table 1, Figure S1A in Supplementary Materials). Similarly, repeated daily oral dose (72 h) mycotoxin treatment with the dose levels of 1 or 10 mg/kg bw also significantly increased the OTA concentration in the blood plasma (Table 1, Figure S1B in Supplementary Materials). In the case of the repeated daily oral dose (21 days; (0.5 mg/kg bw), OTA treatment resulted in a significantly higher OTA level in the blood plasma (Table 1, Figure S1C in Supplementary Materials).

### 2.3. Effect of OTA on the Weight of Spleen and Kidney

Spleen and kidney weight are both indicators of OTA toxicity, but their absolute weight depends on body weight; therefore, organ weights were normalized to the body weight of animals. The single oral OTA treatment (24 h) significantly elevated the normalized wet weight of the kidney in both OTA doses, but the normalized wet weights of the spleen were significantly higher only in the higher OTA-dose-treated group (10 mg/kg bw) (Figure S2A,B in Supplementary Materials). The repeated daily oral dose (72 h) OTA toxicity did not alter the normalized wet weight of the kidney, but the normalized wet weights of the spleen were significantly lower in both MMS and OTA-treated groups, as compared to the control (Figure S3A,B in Supplementary Materials). On the other hand, the repeated daily oral dose (21 days) OTA treatment with the daily dose of 0.5 mg/kg bw decreased the normalized wet weight of the kidney significantly only in the OTA-treated group, as compared to MMS and control groups (Figure S4A in Supplementary Materials). The normalized wet weight of the spleen did not differ significantly between the MMS- and OTA-treated groups (Figure S4B in Supplementary Materials).

### 2.4. Histopathological Analysis of the Renal Cortex

The single oral dose (24 h) OTA expositions did not induce degenerative changes at the histopathological level (data not shown). Repeated daily oral dose (72 h) OTA treatment (1 mg/kg bw or 10 mg/kg bw) showed degenerative lesions predominantly in the inner part of the cortex. Sporadic cell necrosis of the tubular epithelium and cell detachment to the lumen of the tubule were also found. Multifocal necrosis in the tubular cells also occurred. Cell size reduction dispersed apoptotic bodies, and condensed chromatin in the nucleus was observed in the groups treated with high OTA dose and repeated daily oral doses (21 days). However, tubular cell regeneration has also been found in the repeated daily oral dose (21 days) OTA-treated group (Figure S5 in Supplementary Materials).

### 2.5. Changes of Some Parameters of the Glutathione Redox System and Lipid Peroxidation 

**Figure 1:**
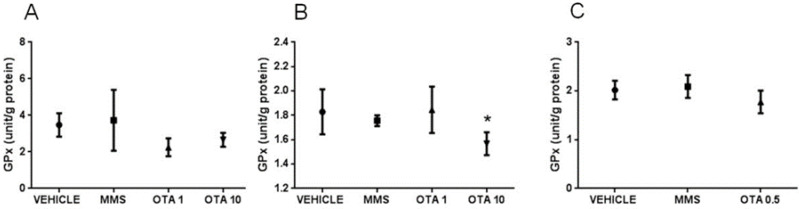
Effect of OTA expositions on GPx activity in the kidney cortex. (**A**) GPx activity of kidney samples in case of single oral dose (24 h) OTA treatment. The applied OTA doses did not change significantly the GPx activity. (**B)** GPx activity of kidney samples in case of repeated daily oral dose (72 h) OTA treatment. The highest applied OTA dose decreased significantly (*p* < 0.05) the GPx activity. (**C**) GPx activity of kidney samples in case of repeated daily oral dose (21 days) OTA treatment. The applied OTA dose did not change the GPx activity significantly. Abbreviations: MMS: methyl-methanesulfonate-treated group; OTA 1 and OTA 10: treatment with 1 and 10 mg/kg bw ochratoxin A in the single oral dose (24 h) and repeated daily oral dose (72 h); OTA 0.5: 0.5 mg/kg bw ochratoxin A treatment in the repeated daily oral dose (21 days) experiment. Mean ± S.D. Data were analyzed by one-way ANOVA and Tukey’s post hoc test.* *p* < 0.05 vs. vehicle.

No differences in glutathione peroxidase (GPx) activity were found on day 1, while in the case of the highest dose of OTA (10 mg/kg bw), significantly (*p* < 0.05) lower GPx activity was found at day 3 in the repeated daily oral dose (72 h) experiment (Figure 1A,B). At the end of the repeated daily oral dose (21 days) OTA treatment, no significant changes were found in GPx activity of kidney samples (Figure 1C).

**Figure 2:**
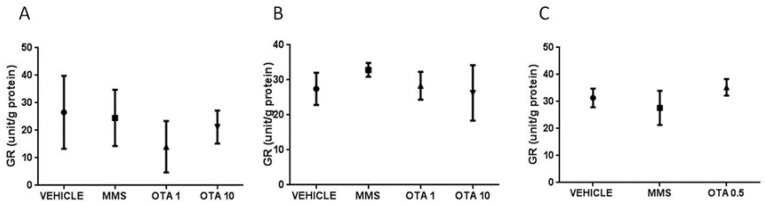
Effect of OTA expositions on the glutathione reductase (GR) activity in the kidney cortex. (**A**) GR activity of kidney samples in case of single oral dose (24 h) OTA treatment. The applied OTA doses did not change significantly the GR activity. (**B**) GR activity of kidney samples in case of repeated daily oral dose (72 h) OTA treatment. The applied OTA doses did not change significantly the GR activity. (**C**) GR activity of kidney samples in case of repeated daily oral dose (21 days) OTA treatment. The applied OTA dose did not change the GR activity significantly. Abbreviations: MMS: methyl-methanesulfonate-treatment group; OTA 1 and OTA 10: 1 and 10 mg/kg bw ochratoxin A treatment in the single oral dose (24 h) and repeated daily oral dose (72 h) groups; OTA 0.5: 0.5 mg/kg bw ochratoxin A treatment in the repeated daily oral dose (21 days) group. Mean ± S.D. Data were analyzed by one-way ANOVA and Tukey’s post hoc test. * *p* < 0.05 vs. vehicle.

Glutathione reductase (GR) activity did not change in the case of a single oral dose (24 h) and repeated daily oral dose (72 h) treatments (Figure 2A,B). In the case of repeated daily oral dose (21 days) treatment, the GR activity also remained unchanged (Figure 2 C).

**Figure 3:**
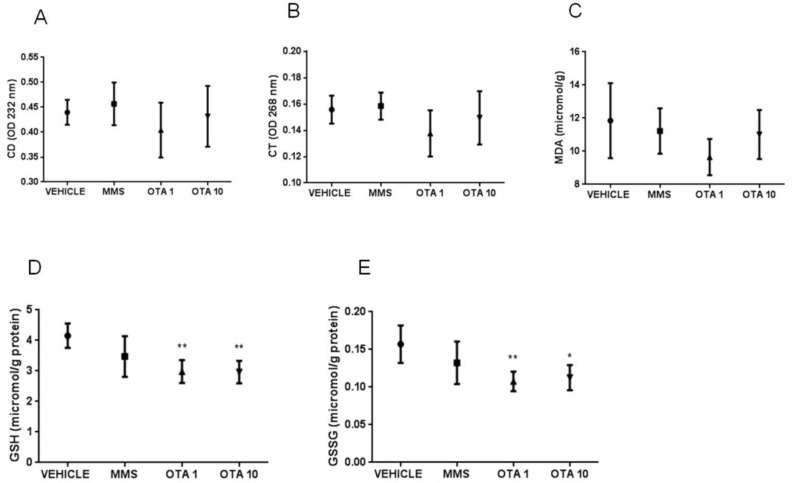
Effect of single oral dose (24 h) OTA exposure on lipid peroxidation parameters and reduced and oxidized glutathione concentration in the kidney cortex. (**A**) Levels of conjugated dienes (CD) in kidney samples in case of single oral dose (24 h) OTA treatment. The applied OTA doses did not cause significant alterations. (**B**) Levels of conjugated trienes (CT) in kidney samples in case of single oral dose (24 h) OTA treatment. The applied OTA doses did not cause significant alteration. (**C**) Malondialdehyde (MDA) concentration in kidney samples in case of single oral dose (24 h) OTA treatment. The applied OTA doses did not cause significant changes. (**D**) Reduced glutathione (GSH) concentration in kidney samples in case of single oral dose (24 h) OTA treatment. Both applied OTA doses decreased significantly (*p* < 0.01) the GSH concentration. (**E**) Oxidized glutathione (GSSG) concentration in kidney samples in case of single oral dose (24 h) OTA treatment. Both applied OTA doses decreased (*p* < 0.01 and *p* < 0.05, respectively) the GSSG concentration significantly. Abbreviations: MMS: methyl-methanesulfonate-treatment. OTA 1 and OTA 10: 1 and 10 mg/kg bw ochratoxin A-treated groups. Mean ± S.D. Data were analyzed by one-way ANOVA and Tukey’s post hoc test. * *p* < 0.05, ** *p* < 0.01 vs. vehicle.

In the case of single oral dose (24 h) OTA treatment, neither the initial phase markers of lipid peroxidation (CD and CT) nor the meta-stable end products (thiobarbituric reactive substances, malondialdehyde (MDA)) showed significant changes (Figure 3A–C).

**Figure 4:**
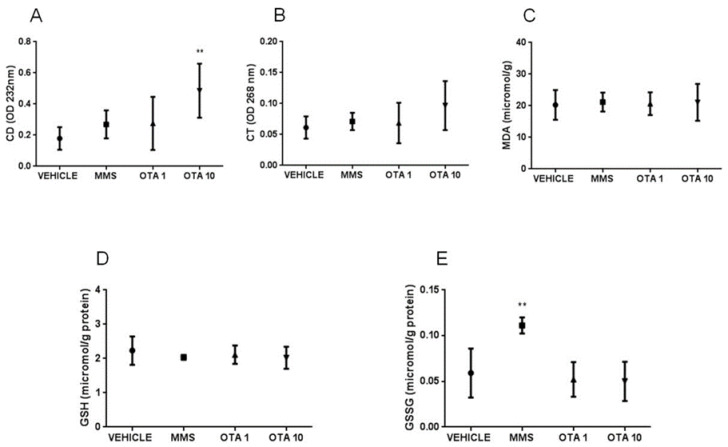
The effect of repeated daily oral dose (72 h) OTA exposition on some lipid peroxidation parameters and reduced and oxidized glutathione concentration in the kidney cortex. **A**: Levels of conjugated dienes (CD) in kidney samples in case of repeated daily oral dose (72 h) OTA treatment. The highest OTA dose increased significantly (*p* < 0.01) the level of conjugated dienes in the kidney. **B**: Levels of conjugated trienes (CT) in kidney samples in case of repeated daily oral dose (72 h) OTA treatment. The applied OTA doses did not cause significant alterations. **C**: Malondialdehyde (MDA) concentration in kidney samples in case of repeated daily oral dose (72 h) OTA treatment. The applied OTA doses did not cause significant changes. **D**: Reduced glutathione (GSH) concentration in kidney samples in case of repeated daily oral dose (72 h) OTA treatment. The applied OTA doses did not cause significant alterations. **E**: Oxidized glutathione (GSSG) concentration in kidney samples in case of repeated daily oral dose (72 h) OTA treatment. The applied OTA doses did not significantly alter, while the MMS treatment increased the GSSG concentration significantly (*p* < 0.01). Abbreviations: MMS: methyl-methanesulfonate-treated group; OTA 1 and OTA 10: 1 and 10 mg/kg bw ochratoxin-A-treated groups. Mean ± S.D. Data were analyzed by one-way ANOVA and Tukey’s post hoc test. ** *p* < 0.01 vs. vehicle.

In case of repeated daily oral dose (72 h) treatment, markers of the early phase of lipid peroxidation increased as a result of high OTA intake (10 mg/kg bw), which was proven by the significant increase in the level of conjugated dienes (CD) at day 3 (*p* < 0.01, Figure 4A). However, the level of conjugated trienes (CT) did not show significant alterations during repeated daily oral dose (72 h) treatment (Figure 4B). On the contrary, lipid peroxidation did not reach the terminal phase in repeated daily oral dose (72 h) treatment, as the thiobarbituric reactive substances were measured by the meta-stable end products (MDA) of this process (Figure 4C).

**Figure 5:**
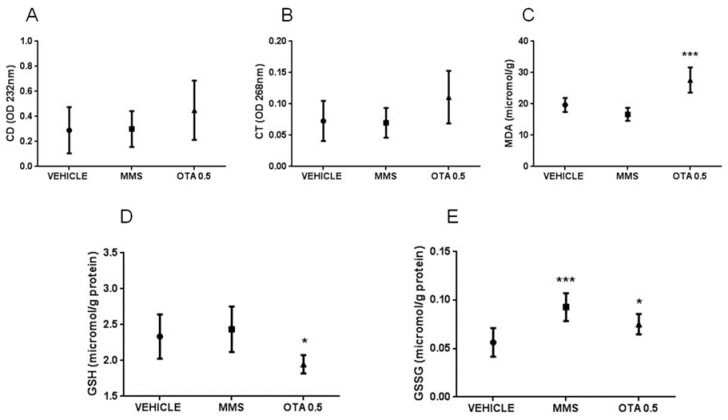
Effect of repeated daily oral dose (21 days) OTA exposition on lipid peroxidation parameters and reduced and oxidized glutathione concentration in the kidney cortex. **A:** Levels of conjugated dienes (CD) in kidney samples in case of repeated daily oral dose (21 days) OTA treatment. The applied OTA dose did not cause significant alteration. **B**: Levels of conjugated trienes (CT) in kidney samples in case of repeated daily oral dose (21 days) OTA treatment. The applied OTA dose did not cause significant alteration. **C**: Malondialdehyde (MDA) concentration in kidney samples in case of repeated daily oral dose (21 days) (21 days) OTA treatment. The applied OTA dose increased the MDA concentration in the kidney significantly (*** *p* < 0.001). **D**: Reduced glutathione (GSH) concentration in kidney samples in case of repeated daily oral dose (21 days) OTA treatment. The applied OTA dose decreased significantly (* *p* < 0.05), the GSH concentration in the kidney. **E**: Oxidized glutathione (GSSG) concentration in the case of kidney samples in repeated daily oral dose (21 days) OTA treatment. The MMS treatment and the applied OTA dose increased significantly (*** *p* < 0.001 and * *p* < 0.05, respectively) the GSSG concentration in the kidney. Abbreviations: MMS: methyl-methanesulfonate-treated group; OTA 0.5: 0.5 mg/kg bw ochratoxin-A-treated group. Mean ± S.D. Data were analyzed by one-way ANOVA and Tukey’s post hoc test. * *p* < 0.05, *** *p* < 0.001 vs. vehicle.

At the end of the repeated daily oral dose (21 days) treatment, no significant changes were measured in the level of CDs and CTs (Figure 5A,B), but the concentration of thiobarbituric acid reactive substances (MDA) was significantly (*p* < 0.001, Figure 5C) higher in the kidney of OTA-treated animals than the controls.

The non-enzymatic components of the glutathione redox system, GSH, and its oxidized form glutathione disulfide (GSSG) concentrations were significantly changed as the effect of both single oral dose (24 h) and repeated daily oral dose (21 days) exposures. GSH concentration in the kidney was lower at day 1 of single oral dose (24 h) OTA exposure in both (1 and 10 mg/kg bw) treated groups (Figure 3D, *p* < 0.01), while at day 3, no significant differences were found (Figure 4D). Repeated daily oral dose (21 days) OTA exposure for 21 days resulted in significantly lower GSH concentration than control (Figure 5D, *p* < 0.05). GSSG concentrations in the kidney were also significantly lower on day 1 (Figure 5E, *p* < 0.01 and *p* < 0.05), but not on day 3 of repeated daily oral dose (72 h) OTA exposure at both doses (Figure 4E). On the contrary, it was significantly higher at the end of the repeated daily oral dose (21 days) OTA exposure (day 21) (Figure 5E, *p* < 0.05). However, on day 3 of the repeated daily oral dose (72 h) experiment, and also at the end (day 21) of the repeated daily oral dose (21 days) treatment, the concentration of GSSG was significantly higher in the MMS group than control (Figure 4E and Figure 5E, *p* < 0.01 and *p* < 0.001, respectively).

### 2.6. Gene Expression of Some Parameters of the Glutathione Redox System

**Figure 6:**
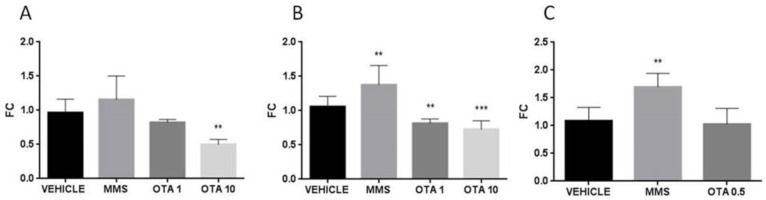
Effect of OTA expositions on the *Gsta* gene expression in the kidney cortex. **A**: *Gsta* gene expression alterations of the single oral dose (24 h) OTA-treated kidney samples. The highest OTA dose decreased the *Gsta* mRNA level (*p* < 0.01). **B**: *Gsta* gene expression alterations of the repeated daily oral dose (72 h) OTA-treated kidney samples. Both applied OTA doses decreased the *Gsta* mRNA levels (*p* < 0.01). **C**: *Gsta* gene expression alterations of the repeated daily oral dose (21 days) OTA-treated kidney samples. OTA treatment did not influence the *Gsta* mRNA level. Abbreviations: MMS: methyl-methanesulfonate-treated group; OTA 1 and OTA 10: 1 and 10 mg/kg bw ochratoxin-A-treated groups in the single oral dose (24 h) and repeated daily oral dose (72 h) experiment; OTA 0.5: 0.5 mg/kg bw ochratoxin-A-treated group in the repeated daily oral dose (21 days) experiment. Mean ± S.D. Data were analyzed by one-way ANOVA and Tukey’s post hoc test. * *p* < 0.05, ** *p* < 0.01, *** *p* < 0.001 vs. vehicle.

In the case of the single oral dose (24 h) and repeated daily oral dose (72 h) treatments, the applied higher dose of OTA decreased the expression of glutathione S- transferase (*Gsta*) on day 1 and also at day 3 of the experiment significantly (Figure 6A,B, *p* < 0.01), and the lower dose of OTA resulted in significantly lower expression of *Gsta* at day 3 of the experiment (*p* < 0.01) (Figure 6B). In the case of the single oral dose (24 h) OTA treatment, the *Gsta* expression was significantly lower than control (Figure 6A *p* < 0.01). However, repeated daily oral dose (21 days) OTA treatment did not cause significant alteration in *Gsta* expression (Figure 6C). In the case of MMS treatment, at day 3 of the repeated daily oral dose (72 h) experiment, significantly (*p* < 0.01) elevated *Gsta* expression was found than in the control group (vehicle) (Figure 6B).

**Figure 7:**
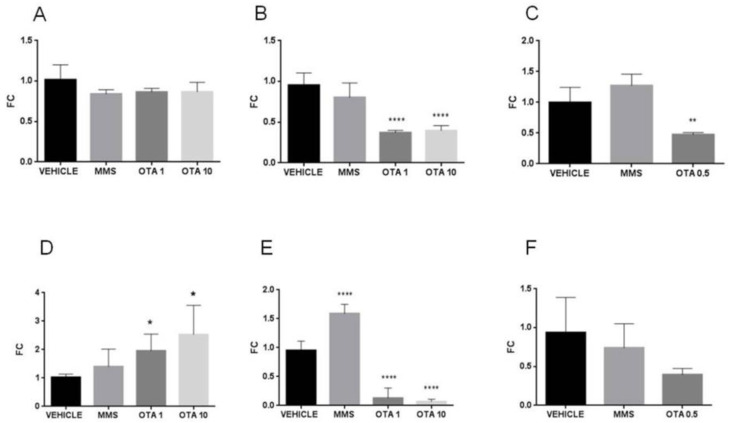
Effect of OTA expositions on the *Gpx1* and *Gpx2* mRNA expression levels in the kidney cortex. **A**: *Gpx1* gene mRNA expressions of the single oral dose (24 h) OTA-treated kidney samples. OTA treatment did not influence the *Gpx1* mRNA level. **B**: *Gpx1* gene expression levels of the repeated daily oral dose (72 h) OTA-treated kidney samples. Both applied OTA doses decreased the *Gpx1* mRNA levels (*p* < 0.001). **C:** *Gpx1* gene expression alterations of the repeated daily oral dose (21 days) OTA-treated kidney samples. The applied OTA dose decreased the *Gpx1* mRNA level (*p* < 0.01). **D**: *Gpx2* gene expression of the single oral dose (24 h) OTA-treated kidney samples. Both applied OTA doses increased the *Gpx2* mRNA levels (*p* < 0.05). **E**: *Gpx2* gene expression alterations of the repeated daily oral dose (72 h) OTA-treated kidney samples. Both applied OTA doses decreased the *Gpx1* mRNA levels (*p* < 0.001). **F**: *Gpx2* gene expression levels of the repeated daily oral dose (21 days) OTA-treated kidney samples. OTA treatment did not significantly influence the *Gpx2* mRNA level. Abbreviations: MMS: methyl-methanesulfonate-treated group; OTA 1 and OTA 10: 1 and 10 mg/kg bw ochratoxin-A-treated groups in the single oral dose (24 h) and repeated daily oral dose (72 h) experiment; OTA 0.5: 0.5 mg/kg bw ochratoxin-A-treated group in the repeated daily oral dose (21 days) experiment. Mean ± S.D. Data were analyzed by one-way ANOVA and Tukey’s post hoc test. * *p* < 0.05, ** *p* < 0.01, **** *p* < 0.0001 vs. vehicle.

Glutathione peroxidase 1 (*Gpx1*) expression in case of single oral dose (24 h) treatment did not change at day 1, but at day 3, both applied doses caused significantly lower expression (*p* < 0.0001) than the control (vehicle) (Figure 7A,B), and at day 21 (*p* < 0.01) of the repeated oral dose (Figure 7C). As regards glutathione peroxidase 2 (*Gpx2*) expression, significant (*p* < 0.05) elevation was found in both treated groups on day 1, while a significant (*p* < 0.0001) decrease was observed thereafter, at day 3, of OTA treatment (Figure 7D,E).

### 2.7. Expression Alteration at mRNA and Protein Level of Nrf2 and Keap1 

**Figure 8:**
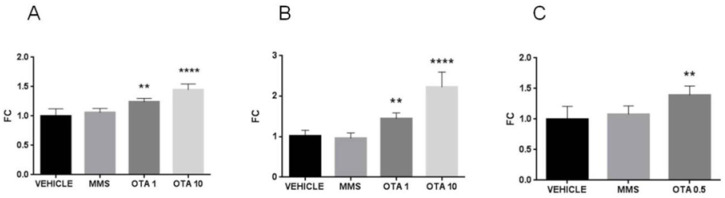
Effect of OTA expositions on the *Nrf2* mRNA expression levels in the kidney. (**A**) *Nrf2* gene mRNA expressions of the single oral dose (24 h) OTA-treated kidney samples. Both applied OTA doses increased the *Nrf2* mRNA levels (*p* < 0.01 and 0.0001, respectively). (**B**) *Nrf2* gene expression levels of the repeated daily oral dose (72 h) OTA-treated kidney samples. Both applied OTA doses increased the *Nrf2* mRNA levels (*p* < 0.01 and 0.0001, respectively). (**C**) *Nrf2* gene expression alterations of the repeated daily oral dose (21 days) OTA-treated kidney samples. The applied OTA dose increased the *Nrf2* mRNA level (** *p* < 0.01). Abbreviations: MMS: methyl-methanesulfonate-treated group; OTA 1 and OTA 10: 1 and 10 mg/kg bw ochratoxin-A-treated groups in the single oral dose (24 h) and repeated daily oral dose (72 h) experiment; OTA 0.5: 0.5 mg/kg bw ochratoxin-A-treated group in the repeated daily oral dose (21 days) experiment. Mean ± S.D. Data were analyzed by one-way ANOVA and Tukey’s post hoc test. ** *p* < 0.01, **** *p* < 0.0001 vs. vehicle.

**Figure 9:**
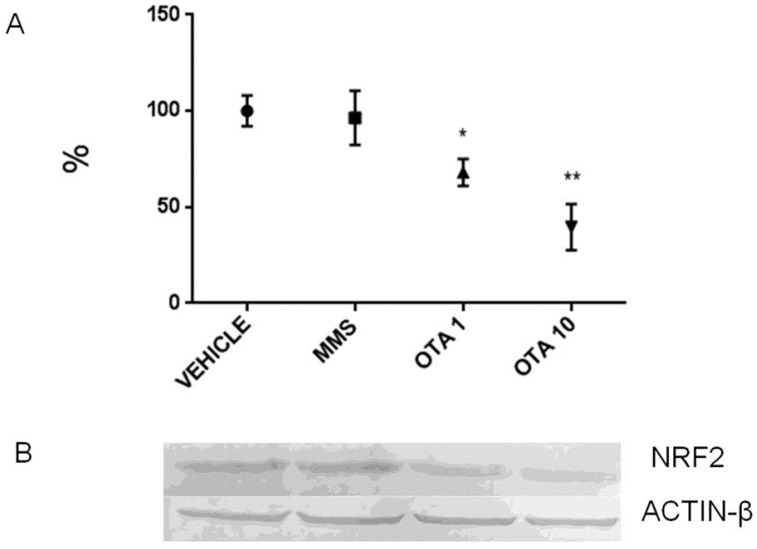
Effect of single oral dose (24 h) OTA expositions on the NRF2 protein expression levels in the kidney. (**A**) Quantification of protein expression normalized to vehicle-treated controls by densitometry. Significant differences were found in the OTA-treated animals. (**B**) Representative examples of Western blots using kidney homogenates from mice. Abbreviations: MMS: methyl-methanesulfonate-treated group; OTA 1 and OTA 10: 1 and 10 mg/kg bw ochratoxin-A-treated groups in the single oral dose (24 h) experiment. Mean ± S.D. Data were analyzed by one-way ANOVA and Tukey’s post hoc test. * *p* < 0.05, ** *p* < 0.01 vs. vehicle.

**Figure 10:**
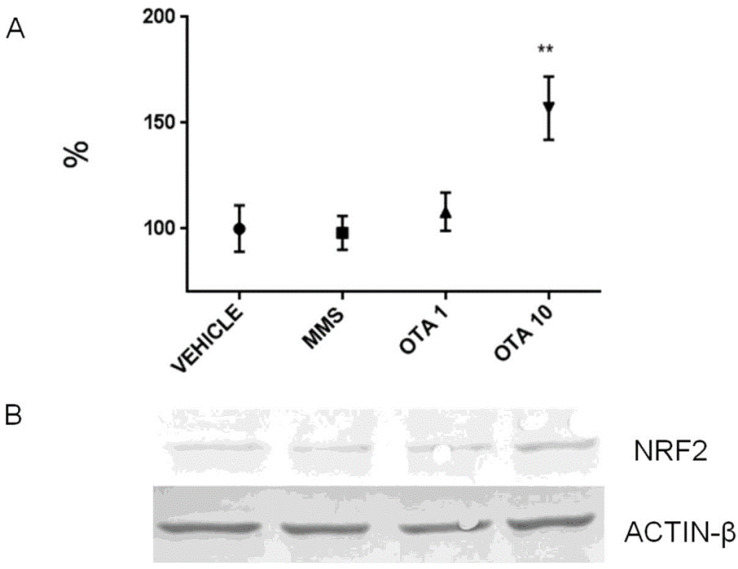
Effect of repeated daily oral dose (72 h) OTA expositions on the expression of NRF2 protein in the kidney. (**A**) Protein expression normalized to vehicle-treated controls by densitometry. Significant protein expression elevation was found in the high dose of OTA-treated animals. (**B**) Representative examples of Western blots from kidney homogenates mice. Abbreviations: MMS: methyl-methanesulfonate-treated group; OTA 1 and OTA 10: 1 and 10 mg/kg bw ochratoxin-A-treated groups in the repeated daily oral dose (72 h) experiment. Mean ± S.D. Data were analyzed by one-way ANOVA and Tukey’s post hoc test. ** *p* < 0.01 vs. vehicle.

**Figure 11:**
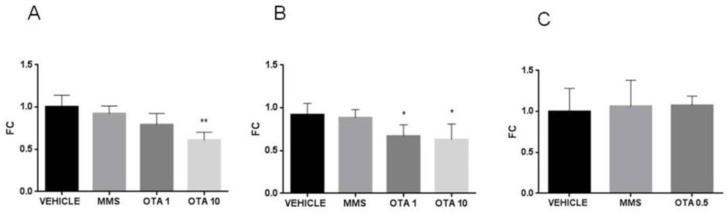
Effect of OTA expositions on the *Keap1* mRNA expression levels in the kidney. **A**: *Keap1* gene mRNA expressions of the single oral dose (24 h) OTA-treated kidney samples. The highest OTA dose decreased the *Keap1* mRNA level (*p* < 0.01). **B**: *Keap1* gene expression levels of the repeated daily oral dose (72 h) OTA-treated kidney samples. Both applied OTA doses decreased the *Keap1* mRNA levels (*p* < 0.05). **C**: *Keap1* gene expression alterations of the repeated daily oral dose (21 days) OTA-treated kidney samples. The OTA treatment did not influence the *Keap1* mRNA level significantly. Abbreviations: MMS: methyl-methanesulfonate-treated group, OTA 1; and OTA 10: 1 and 10 mg/kg bw ochratoxin-A-treated groups in the single oral dose (24 h) and repeated daily oral dose (72 h) experiment; OTA 0.5: 0.5 mg/kg bw ochratoxin-A-treated group in the repeated daily oral dose (21 days) experiment. Mean ± S.D. Data were analyzed by one-way ANOVA and Tukey’s post hoc test.* *p* < 0.05, ** *p* < 0.01 vs. vehicle.

Single oral dose (24 h) OTA treatment significantly increased the *Nrf2* expression at mRNA level at both OTA doses (Figure 8A, *p* < 0.01 and *p* < 0.001, respectively). However, the NRF2 protein level was robustly decreased at both OTA doses introduced (*p* < 0.05 in the OTA 1 group and *p* < 0.01 in the OTA 10 group) (Figure 9). However, the phosphorylated NRF2 (at Ser40 amino acid) protein level was not changed due to OTA administration (Figure S6 in Supplementary Materials). The NRF2 protein level was increased at the higher OTA-treated group (*p* < 0.01) (Figure 10). On the other hand, the Ser40 phosphorylated NRF2 expressions remained stable at the protein level (Figure S7 in Supplementary Materials). The expression of the Keap1 significantly decreased at mRNA level by dose-dependent manner to single oral dose (24 h) OTA administration on day 1 (Figure 11, *p* < 0.01 at the highest OTA-dose-treated group). On day 3, the repeated daily oral dose (72 h) OTA administration elevated the *Nrf2* mRNA level at both applied doses (*p* < 0.01 in the OTA 1 group and *p* < 0.001 in the OTA 10 group) (Figure 8B). On day 3 of OTA exposure, the *Keap1* mRNA level decreased significantly, but independently of the OTA dose applied (Figure 11B, *p* < 0.05). The repeated daily oral dose (21 days) OTA treatment significantly elevated the *Nrf2* mRNA level (Figure 8C, *p* < 0.01), but the protein levels of NRF2 and its Ser40 phosphorylated form were not altered (Figures S8 and S9 in Supplementary Materials). The repeated daily oral dose (21 days) administration of the MMS as a positive control caused an increase in the NRF2 and NRF2 (Ser40) phosphorylated protein levels (Figures S8 and S9, *p* < 0.01). However, the repeated daily oral dose (21 days) OTA treatment did not influence the *Keap1* mRNA expression (Figure 11C).

### 2.8. Gene Expression Alteration of the Antioxidant Response Element (ARE) Responsive Genes

**Figure 12:**
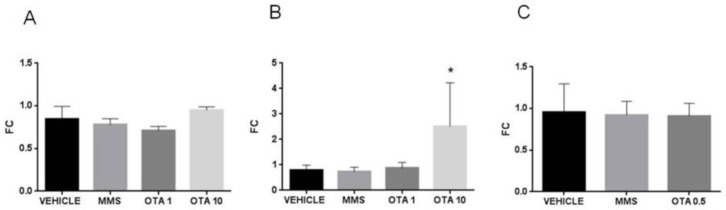
Effect of OTA expositions on the hem-oxygenase (*Ho-1*) gene expression levels in the kidney. **A**: *Ho-1* gene mRNA expressions of the single oral dose (24 h) OTA-treated kidney samples. The OTA treatment had no significant effect on the *Ho-1* mRNA level. **B**: *Ho-1* gene expression levels of the repeated daily oral dose (72 h) OTA-treated kidney samples. The high dose of OTA treatment increased the *Ho-1* mRNA level significantly (*p* < 0.05). **C**: *Ho-1* gene expression alterations of the repeated daily oral dose (21 days) OTA-treated kidney samples. The OTA treatment did not influence significantly the *Ho-1* mRNA level. Abbreviations: MMS: Group treated with methyl-methanesulfonate; OTA 1 and OTA 10: groups treated with 1 and 10 mg/kg bw ochratoxin A in the single oral dose (24 h) and repeated daily oral dose (72 h) experiment; OTA 0.5: group treated with 0.5 mg/kg bw ochratoxin A in the repeated daily oral dose (21 days) experiment. Values are expressed as the mean ± SD. Data were analyzed by one-way ANOVA followed by Tukey’s post hoc test.* *p* < 0.05 vs. vehicle.

**Figure 13:**
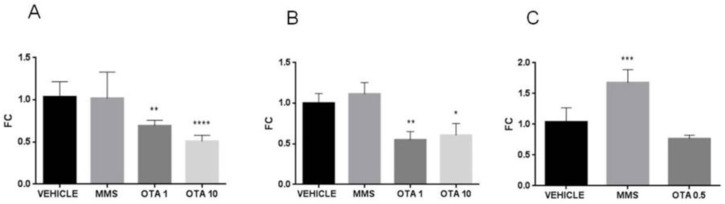
Effect of OTA expositions on the *Nqo1* mRNA expression levels in the kidney. **A**: *Nqo1* gene mRNA expressions of the single oral dose (24 h) OTA-treated kidney samples. Both applied OTA doses decreased the *Nqo1* mRNA levels (*p* < 0.01 and *p* < 0.001, respectively). **B**: *Nqo1* gene expression levels of the repeated daily oral dose (72 h) OTA-treated kidney samples. Both applied OTA doses decreased the *Nqo1* mRNA levels (*p* < 0.01, and *p* < 0.05, respectively). **C**: *Nqo1* gene expression alterations of the repeated daily oral dose (21 days) OTA-treated kidney samples. The OTA treatment did not have a significant effect on the *Nqo1* mRNA level. Abbreviations: MMS: methyl-methanesulfonate-treated treated group; OTA 1 and OTA 10: 1 and 10 mg/kg bw ochratoxin-A-treated groups in the single oral dose (24 h) and repeated daily oral dose (72 h) experiment; OTA 0.5: 0.5 mg/kg bw ochratoxin-A-treated group in the repeated daily oral dose (21 days) experiment. Mean ± S.D. Data were analyzed by one-way ANOVA and Tukey’s post hoc test.* *p* < 0.05, ** *p* < 0.01, *** *p* < 0.001, **** *p* < 0.0001 vs. vehicle.

**Figure 14:**
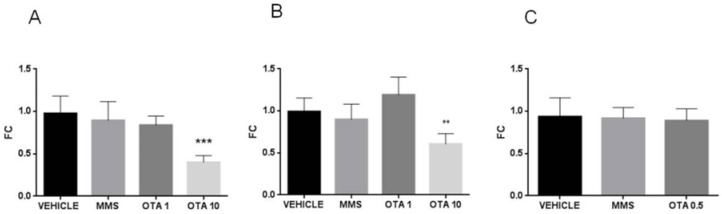
Effect of OTA expositions on the *Gss* mRNA expression levels in the kidney. **A**: *Gss* gene mRNA expressions of the single oral dose (24 h) OTA-treated kidney samples. The highest OTA dose decreased the *Gss* mRNA levels (*p* < 0.001). **B**: *Gss* gene expression levels of the repeated daily oral dose (72 h) OTA-treated kidney samples. The highest OTA dose decreased the *Gss* mRNA levels (*p* < 0.01). **C**: *Gss* gene expression alterations of the repeated daily oral dose (21 days) OTA-treated kidney samples. The applied OTA dose did not cause significant alteration. Abbreviations: MMS: methyl-methanesulfonate-treated group; OTA 1 and OTA 10: 1 and 10 mg/kg bw ochratoxin-A-treated groups in the single oral dose (24 h) and repeated daily oral dose (72 h) experiment; OTA 0.5: 0.5 mg/kg bw ochratoxin-A-treated group in the repeated daily oral dose (21 days) experiment. Mean ± S.D. Data were analyzed by one-way ANOVA and Tukey’s post hoc test. ** *p* < 0.01, *** *p* < 0.001 vs. vehicle.

The heme oxygenase 1 (*Ho-1*) mRNA level increased significantly in repeated daily oral dose (72 h) toxicity at the highest dose of mycotoxin exposure exclusively (Figure 12B, *p* < 0.05), but did not change as effect of repeated oral dose for 21 days (Figure 12C). The NAD(*p*)H dehydrogenase (quinone) (*Nqo1*) mRNA levels reduced significantly in the single oral dose (24 h) and repeated daily oral dose (72 h) OTA-treated animals, independently of the applied OTA dose (Figure 13A,B, *p* < 0.01, *p* < 0.001 and *p* < 0.01, *p* < 0.05, respectively). However, repeated daily oral dose (21 days) OTA exposure did not affect *Nqo1* gene expression (Figure 13C). The glutathione synthetase (*Gss*) mRNA expression was reduced exclusively in a single oral dose (24 h) and repeated daily oral dose (72 h) experiments in a dose-dependent manner (Figure 14A,B, *p* < 0.001, and *p* < 0.01, respectively), but no significant changes were found as effect of repeated oral dose for 21 days (Figure 14C).

### 2.9. Expression Changes of the Duperoxide Dismutase (Sod) Enzymes in the Kidney

**Figure 15:**
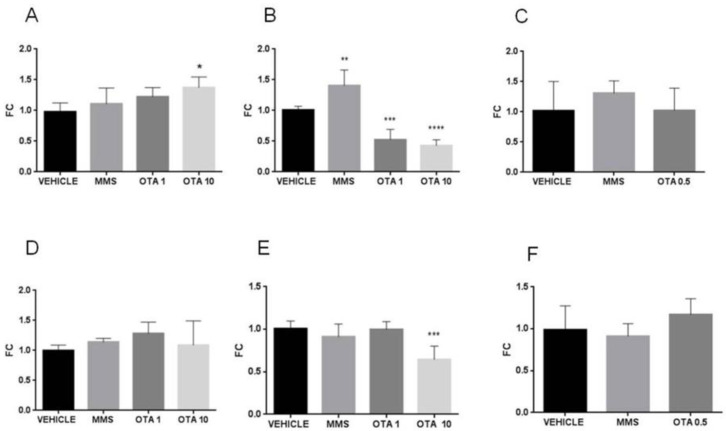
Effect of OTA expositions on the *Sod1* and *Sod2* mRNA expression levels in the kidney. **A**: *Sod1* gene mRNA expressions of the single oral dose (24 h) OTA-treated kidney samples. The high OTA dose significantly increased the *Sod1* mRNA level (*p* < 0.05). **B**: *Sod1* gene expression levels of the repeated daily oral dose (72 h) OTA-treated kidney samples. Both OTA administration significantly decreased the *Sod1* mRNA level (*p* < 0.001 and 0.0001, respectively). **C**: *Sod1* gene expression alterations of the repeated daily oral dose (21 days) OTA-treated kidney samples. The OTA treatment did not significantly influence the *Sod1* mRNA level. **D**: *Sod2* gene mRNA expressions of the single oral dose (24 h) OTA-treated kidney samples. The OTA treatment did not significantly influence the *Sod2* mRNA level. **E**: *Sod2* gene expression levels of the repeated daily oral dose (72 h) OTA-treated kidney samples. The high OTA dose treatment significantly decreased the *Sod2* mRNA level (*p* < 0.001), **F**: *Sod2* gene expression alterations of the repeated daily oral dose (21 days) OTA-treated kidney samples. The OTA treatment did not significantly influence the *Sod2* mRNA level. Abbreviations: MMS: methyl-methanesulfonate-treated group; OTA 1 and OTA 10: 1 and 10 mg/kg bw ochratoxin-A-treated groups in the single oral dose (24 h) and repeated daily oral dose (72 h) experiment; OTA 0.5: 0.5 mg/kg bw ochratoxin-A-treated group in the repeated daily oral dose (21 days) experiment. Mean ± S.D. Data were analyzed by one-way ANOVA and Tukey’s post hoc test * *p* < 0.05, ** *p* < 0.01, *** *p* < 0.001, **** *p* < 0.0001 vs. vehicle.

Single OTA administration significantly increased *Sod1* mRNA expression at the highest dose treated group (Figure 15A, *p* < 0.05), but not significant difference was found in *Sod2* mRNA expression (Figure 15D). However, at day 3, after repeated daily oral dose (72 h), OTA exposure in both enzyme *Sod1* and *Sod2* expressions at mRNA level decreased significantly (Figure 15B, *p* < 0.01 and *p* < 0.001 in OTA 1 and OTA 10 groups, respectively) (Figure 15E, *p* < 0.001 in OTA 10 group). Repeated daily oral dose (21 days) mycotoxin treatment did not influence the Sod enzymes at the mRNA level significantly (Figure 15C,F).

### 2.10. Hace1 and Rac1 mRNA Expression Changes by OTA Administration in the Kidney

**Figure 16:**
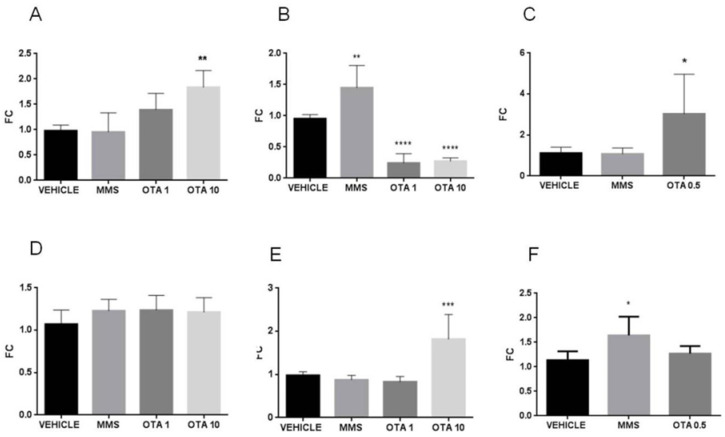
Effect of OTA expositions on the *Hace1* and *Rac1* mRNA expression levels in the kidney. **A**: *Hace1* gene mRNA expressions of the single oral dose (24 h) OTA-treated kidney samples. The high OTA dose significantly increased the *Hace1* mRNA level (*p* < 0.01). **B**: *Hace1* gene expression levels of the repeated daily oral dose (72 h) OTA-treated kidney samples. Both OTA administrations significantly decreased the *Hace1* mRNA level (*p* < 0.0001). **C**: *Hace1* gene expression alterations of the repeated daily oral dose (21 days) OTA-treated kidney samples. The OTA treatment significantly increased the *Hace1* mRNA level (*p* < 0.05). **D**: *Rac1* gene mRNA expressions of the single oral dose (24 h) OTA-treated kidney samples. The OTA treatment did not significantly influence the *Rac1* mRNA level. **E**: *Rac1* gene expression levels of the repeated daily oral dose (72 h) OTA-treated kidney samples. The high OTA dose treatment significantly increased the *Rac1* mRNA level (*p* < 0.001), **F**: *Rac1* gene expression alterations of the repeated daily oral dose (21 days) OTA-treated kidney samples. The OTA treatment did not significantly influence the *Rac1* mRNA level. Abbreviations: MMS: methyl-methanesulfonate-treated group; OTA 1 and OTA 10: 1 and 10 mg/kg bw ochratoxin-A-treated groups in the single oral dose (24 h) and repeated daily oral dose (72 h) experiment; OTA 0.5: 0.5 mg/kg bw ochratoxin-A-treated group in the repeated daily oral dose (21 days) experiment. Mean ± S.D. Data were analyzed by one-way ANOVA and Tukey’s post hoc test.* *p* < 0.05, ** *p* < 0.01, *** *p* < 0.001, **** *p* < 0.0001 vs. vehicle.

Single high-dose OTA administration increased the HECT domain. The mRNA expression of the ankyrin repeat-containing E3 ubiquitin-protein ligase 1 (*Hace1*) also increased significantly (Figure 16A, *p* < 0.01). On the other hand, the repeated daily oral dose (72 h) OTA exposition significantly decreased the *Hace1* expression at the mRNA level in both applied dose (Figure 16B, *p* < 0.001). On the contrary, the repeated daily oral dose (21 days) OTA exposition significantly increased the *Hace1* mRNA level (Figure 16C, *p* < 0.05). The Rac family small GTPase 1 (*Rac1*) mRNA expression significantly elevated at day 3 in kidney samples of the highest OTA-exposed group (Figure 16E, *p* < 0.001). The other treatments did not alter the Rac1 expressions (Figure 16D,F).

## 3. Discussion

The NRF2 and KEAP1, as master regulators of the antioxidant defense, have an effect on the gene expression of the phase II enzymes, such as Heme Oxygenase (HO-1), NQO1, and GSS for cellular protection against oxidative stress [20]. Our in vivo experiments demonstrate that OTA exposition significantly increases the *Nrf2* mRNA levels both in single oral dose (24 h), repeated daily oral dose (72 h), or repeated daily oral dose (21 days) OTA expositions. A similar observation was published in a rat model after a 28 days OTA exposition [16]. However, previous in vitro studies demonstrated on porcine LLC-PK1 kidney cell lines that OTA exposure for 12 and 24 h decreased *Nrf2* mRNA and NRF2 protein levels. Moreover, the presence of NRF2 protein in the nucleus was significantly reduced by the OTA exposition, suggesting that a high OTA dose inhibits the NRF2 translocation into the nucleus [30]. In our in vivo study, the NRF2 protein levels were decreased after a single OTA treatment on day 1 in a dose-dependent manner (Figure 9). NRF2-dependent gene transcriptions, such as *Gss* and *Nqo1*, were significantly decreased at mRNA level, equally in a dose-dependent or -independent manner, respectively (Figure 14A and Figure 13A). Interestingly, the *Ho-1* expression remained stable (Figure 12A) in the present trial. The in vitro 24 h OTA exposition on the LLC-PK1 kidney cell line revealed lower *Ho-1* mRNA and HO-1 protein levels, as well [31]. However, in an in vivo experiment at day 1 of OTA treatment (1 mg/kg bw), a significantly elevated HO-1 protein level was found in toxin-sensitive male Fischer-344 rats [32]. The GSH, the end product of the activity of GSS, was significantly decreased, independently of the applied OTA dose. For this reason, the glutathione synthesis seems to be the most sensitive antioxidant pathway to a single dose of OTA exposition.

HACE1 and RAC1 is the other antioxidant response pathway. A single OTA treatment significantly elevated the *Hace1* mRNA synthesis when the higher OTA dose (10 mg/kg bw) was applied. The lower dose (1 mg/kg bw) and repeated oral treatment for 21 days with a dose of 0.1 mg/kg bw revealed that the *Rac1* expression remained stable.

The highest *Nrf2* mRNA elevation was detected at day 3 in the kidney of OTA-treated animals at both applied doses (1 or 10 mg/kg bw) in a single (24 h) or repeated (72 h) oral exposure. However, the NRF2 protein level was significantly elevated, but GSS and *Nqo1* mRNA levels decreased significantly on day 3 in the treated animals’ kidney. On the other hand, the NRF2-dependent *Ho-1* mRNA level increased considerably in the kidney of the high OTA dose (10 mg/kg bw) in single (24 h) or repeated (72 h) exposed animals. The presence of high OTA content in the kidney cells presumably inhibits the activated NRF2 protein translocation into the nucleus [30], resulting in lower transcription frequency in ARE-related genes such as the *Gss* and *Nqo1*. Altered NRF2 protein distribution in the cytoplasm and the nucleus was detected in an in vitro model in HepG2 cells treated with OTA. For instance, cells treated with OTA for 48 h showed remarkable retention of NRF2 protein in the cytoplasm [33]. The histopathological analysis revealed that the presence of the necrotic tubular cells was more abundant at day 3 in animals exposed to 10 mg OTA/kg bw as compared to treated groups exposed for a shorter period (day 1) and lower (1 mg/kg bw) OTA dose (Suppl. Fig. S5). A similar observation was published in a rat model at day 3 of OTA administration at 10 mg OTA/kg bw dose [13]. However, the 3 days long exposition of high OTA dose induced certain DNA damage, apoptosis, and cancer-related gene expression activation, such as Gadd45, Gadd153, annexin2, and clusterin, served as markers of the single oral dose (24 h) free-radical-mediated tissue damage in the kidney [13,34]. The OTA induced single oral dose (24 h) kidney tubular cell necrosis, presumably activating the HO-1 expression independently of the NRF2 [35]. Toxic agent exposure or injury of the kidney resulted in cellular stress, which destabilizes the intracellular hem proteins, results in ROS formation, and leads to lipid peroxidation [36,37]. As a result of activation of the HO-1, a cellular defense mechanism was induced by the free-radical-mediated tissue damage, but the organism was unable to protect the cells from the harmful effects of the uncontrolled lipid peroxidation in the kidney. A good explanation for this phenomenon is the remarkable elevated level of CD at 10 mg OTA/kg bw treated repeated daily oral dose (72 h) group (Figure 6A), indicating the increase in the initial phase of lipid peroxidation. Inhibition of *Gss* mRNA synthesis and reduced GSS activity at the 10 mg OTA/kg bw treated animals and reduced mRNA levels of *Gpx1* and *Gpx2* at both applied OTA doses led to an unaltered level of the GSH and GSSG in the kidney. Moreover, the *Gsr* expression and GR activity remained stable, which means that the glutathione redox cycle remained more or less unaltered; therefore, it can neutralize ROS and metabolites. This finding is supported by the increase in the initial, but not the terminal, phase markers of lipid peroxidation, which means no chain reaction occurred after initiation.

The repeated daily oral dose (72 h) OTA exposition significantly decreased the *Sod1* and mitochondrial *Sod2* (at high, 10 mg/kg bw, OTA dose only) mRNA expression levels in the kidney. Previous findings in the human embryonic kidney (HEK-293) cells demonstrated that OTA exposure caused ROS overproduction and decreased SOD activity [38]. Moreover, the same study on HEK-293 cells demonstrated a loss of mitochondrial membrane potential [38], suggesting the functional failure of the mitochondrion as an effect of OTA exposure. Inhibition of hydrogen peroxide detoxifying enzymes, such as catalase or glutathione-peroxidase (GPx) in hepatic cell culture by 3-amino-1,2,4-triazole and mercaptosuccinic acid, significantly decreased the SOD2 expression (both at mRNA and protein level) and robustly increased the number of the necrotic cells in an in vitro model [39]. These results are confirmed by the elevated number of the necrotic tubular cells in the repeated daily oral dose (72 h) high-dose-OTA-treated mice.

The *Hace1* mRNA levels were robustly decreased in a dose-independent manner at day 3 of OTA exposition. Moreover, the *Rac1* expression was increased at the 10 mg OTA/kg bw treated group. Hace1 knockout mice showed NADPH oxidase-dependent ROS elevation [29].

The *Rac1* expression increased exclusively when the highest OTA dose was introduced, but then, the ace1 expression change was dose-independent. The elevated level of the *Rac1* suggests the *Nox1-*dependent ROS overproduction at the 10 mg OTA / kg bw treated mice’s kidney. Repeated daily oral dose (21 days) OTA exposure significantly elevated the *Nrf2* mRNA level, but the protein expression remained stable. In addition, the *Keap1* mRNA expression remained stable. Previous studies demonstrated that chronic (6 or 12 months) OTA exposition in a rat model significantly reduces the cellular antioxidant defense by inhibiting NRF2 [18,40]. The OTA-induced oxidative stress did not influence the NRF2 regulated *Gss*, *Nqo1*, and *Ho-1* expressions in our experiments. In a rat model, elevated *Nrf2* and unaltered *Nqo1* and *Ho-1* mRNA levels were observed at day 28 of OTA (0.21 mg/kg bw) exposition in the kidney [16]. The *Hace1* mRNA expression was elevated considerably in the kidney cortex of the repeated daily oral dose (21 days) OTA-treated animals. However, the HACE1 target *Rac1* mRNA level remained unchanged. The activation of the HACE1 suggests the higher frequencies of the RAC1 protein inactivation leads to lower *Nox1*-dependent ROS production. 

OTA is a well-known initiator of oxygen free radical formation in the kidney [41] and, consequently, activate lipid peroxidation [42]. These findings did not support the present study results in a single oral dose (24 h) exposure on day 1. Still, on day 3, the marker of the initial phase of lipid peroxidation, CDs, revealed such a process. The result can be explained with the significant decrease in GSH and simultaneous activation of *Gpx2* gene expression on day 1, which showed the opposite tendency two days later. It means that early activation of some components of the glutathione redox system eliminated the free radicals formed as an effect of OTA exposure, but this effect was eliminated during the next two days; therefore, no adequate antioxidant defense was available, and free radicals were able to initiate lipid peroxidation. Another glutathione redox component, GST, did not activate as the effect of OTA exposure. This is contradictory to some previous data when oxidative stress upregulated GST [43]. Repeated daily oral dose (21 days) (0.5 mg/kg bw) of OTA exposure revealed lipid peroxidation, which reached the terminal phase, as shown by thiobarbituric acid reactive substances concentration, at the end of the 21-days trial. Free radical formation and lipid peroxidation in chronic exposure, supported by the results of [44] in rats, caused a significant decrease in GSH and an increase in GSSG concentrations. The decrease in GSH and increase in GSSG cannot explain with the changes in the activities of GPx and G.R., because those remained at the control level. However, marked and significantly lower expression of both enzymes was found. Previous research also suggests that oxidative stress in response to OTA may result from the down-regulation of genes involved in antioxidant defense [18,40]. These results suggest that probably other mechanisms, such as conjugation of the non-chlorinated metabolite of OTA with GSH [45], may result in its lower concentration.

The NRF2 Ser40-*p* protein level in the kidney was not influenced either in a single oral dose (24 h) or repeated daily oral dose (21 days) OTA exposition. The release of NRF2 from KEAP1 possibly affects PKC-δ-mediated phosphorylation of NRF2 S40-P [46]. This step is essential to the activated NRF2 translocation from the cytoplasm to the nucleus [46]. A previous study demonstrated that KEAP1 controls the ubiquitination of NRF2. This process is mediated by Cullin 3 (Cul3)-dependent ubiquitin ligase (E3) [47]. Lack of the alteration of the Ser40 phosphorylation of the NRF2 at the day 3 OTA exposed samples, which is an essential step for the release of NRF2 from KEAP1 binding, suggests that de novo transcription and translation is the primary mechanism to increase the NRF2 level in the cytoplasm. OTA treatment in itself did not influence NRF2 Ser40 phosphorylation-mediated rescue from degradation. On the other hand, the single OTA treatment robustly reduced NRF2 translation, suggesting their putative post-translation inhibition by microRNAs. A previous study demonstrated a negative regulation of the mir-132 on NRF2 in LLC-PK cells to 24 h of OTA treatment [31]. The role of the microRNAs in the OTA toxicities was not investigated in an in vivo model. The *Keap1* mRNA expression decreased significantly, but the low level at both doses of single oral dose (24 h) OTA exposure, suggesting the diminished rate of NRF2 degradation. The repeated daily oral dose (21 days) OTA administration did not influence the NRF2 Ser40-P protein levels; thus, the reduced antioxidant defense resulted in significant lipid peroxidation, as shown by the concentration of thiobarbituric acid reactive substances.

In conclusion, the results revealed that OTA initiates free radical formation in the kidney. However, this process is more dependent on the duration of exposure than on the dose applied. Free radical formation activates the KEAP1/NRF2/ARE pathway but in a different time and dose-dependent manner. OTA exposition increased the *Nrf2* mRNA levels, suggesting activation of genes encoding ARE, but it was not supported by the NRF2 Ser40-P protein level, which can be transported to the nucleus, and activates ARE. As suggested by the low mRNA level of NRF2-dependent genes, lack of ARE activation resulted in an improper antioxidant defense, thus oxidative stress, and, consequently, cell damage.

## 4. Materials and Methods

### 4.1. Reagents

OTA (Fermentek, Jerusalem, Israel), methyl-methanesulfonate (MMS) (Sigma-Aldrich., St. Louis, MO, USA), dimethyl-sulfoxide-DMSO (Sigma-Aldrich, St. Louis, MO, USA), Tris-(hydroxymethyl)-aminomethane, Tris (Sigma-Aldrich, St. Louis, MO, USA) were used. All other chemicals were obtained from Sigma-Aldrich, USA, at the highest purity as available.

### 4.2. Animals and Diet

Test animals were adult, male CD1 mice (7–9 weeks old) (from the colony breed at the Institute of Experimental Medicine). Animals had free access to feed and tap water. Animals were maintained under controlled conditions (temperature: 21 ± 1 °C; humidity: 65%; dark–light cycle, 12 h dark/12 h light, lights on 07:00). Batches of rodent feedstuff were tested by Soft Flow Hungary R&D Ltd. (Pecs, Hungary), and traces of Ochratoxin-A were detected (3.05 ± 0.12 µg/kg fodder). All procedures and experiments were conducted according to guidelines of the European Communities Council Directive (86/609 EEC), and the protocol was accepted by the Institutional Animal Care and Use Committee of the Institute of Experimental Medicine, Budapest Hungary (permit number: PEI/001/35-4/2013, approval date: 13 February, 2013, this permit is valid for 5 years). Animal experiments were performed in February and March 2013.

### 4.3. Measuring of the OTA Concentration in Blood Plasma by ELISA

OTA extraction was performed from 50 µL plasma samples with 100 µL chloroform and 10µL (0.1 M H_3_PO_4_). The mixture was homogenized and incubated on Bio RS24 vertical mini rotator (bioSan, Riga, Latvia) rotating 25 rpm (20 min), then homogenized and subsequently centrifuged for 10 min (10000× *g*). The lower phase was placed in a plastic tube, mixed with 100 µL (3% (*w/v*)) NaHCO_3_ solution, and subsequently incubated on a Bio RS24 vertical rotator (25 rpm, 10 min). The two bands were obtained, and the upper phase was used for OTA measurements. 

The ochratoxin-A concentration of the extract was determined by Toxi-Watch™ Ochratoxin-A ELISA Kit (Cat#301051, Soft Flow Hungary, Pecs, Hungary). Measurements were carried out on Thermo Scientific Multiskan EX plate absorbance reader (Thermo Scientific, San Jose, CA, USA). Measurements were performed in triplicate.

### 4.4. Animal Treatment

Male, adult (7–9 weeks old) CD1 mice were housed with full access to fodder and tap water. The testing parameters and the applied dose and time were determined, according to Luhe et al. [13] and Zeljezic et al. [48]. Animal experiments were performed in February and March 2013. Bodyweight, food, and tap water consumption were measured daily. Ochratoxin-A was solved in dimethyl-sulfoxide (DMSO), and a master mix in 100 mg/mL OTA content was diluted and stored at 4 °C. This master mix was subsequently diluted with sterile tap water (containing 10 mM Tris, pH 8) to reach the required OTA doses. OTA and vehicle solutions (sterile tap water containing 10 mM Tris, pH 8) were administered once daily via oral gavages (200 µL/animal) in the morning period. 

For testing the toxic effects of OTA, three different dosage groups were established. For a single oral dose (24 h) and repeated daily oral dose (72 h) tests, the groups treated with 1 mg/kg body weight (bw) and 10 mg/kg bw OTA and sacrificed after 24 or 72 h, respectively. Vehicle solution (sterile drinking water contained 10 mM Tris (pH 8)); it was supplemented with the same DMSO amount with which the high OTA dose administration was applied. For the repeated daily oral dose (21 days) (21 days, repeated oral dose) test, the animals treated with 0.5 mg/kg body weight OTA daily were sacrificed on day 21. Sterile tap water (containing 10 mM Tris, pH 8) and equal concentration of dimethyl-sulfoxide with high-dose-OTA-treated groups in the single oral dose (24 h) and repeated daily oral dose (72 h) experiments and equal concentration of DMSO with the repeated daily oral dose (21 days) experiment were applied in the control (vehicle) groups. As a positive reference, MMS treatment was introduced in 100 mg/kg bw (per day) dose for the single oral dose (24 h) and repeated daily oral dose (72 h) experiments and 40 mg/kg body weight dose for the repeated daily oral dose (21 days) experiment—Zeljezic et al. [48]. The testing parameters and the applied dose and time were determined, according to Luhe et al. [13] and Zeljezic et al. [48]. For the single oral dose (24 h) experiment, 6 animals were used in the vehicle group; 6 animals were used in the MMS-treated, positive control group; 7 animals were used in the 1 mg/kg body weight OTA-treated group; 7 animals were used in the 10 mg/kg body weight OTA-treated group. For repeated daily oral dose (72 h) experiment, 6 animals were used in the vehicle group; 6 animals were used in the MMS-treated, positive control group; 10 animals were used in the 1 mg/kg body weight OTA-treated group; 7 animals were used in the 10 mg/kg body weight OTA-treated group. For repeated daily oral dose (21 days) experiment, 8 animals were used in the Vehicle group; 6 animals were used in the MMS-treated and positive control group; 10 animals were used in the 0.5 mg/kg body weight OTA-treated group. 

Animals were decapitated, trunk blood was collected to polypropylene tube (BD Vacutainer K2EDTA Plus Tubes, BD, Franklin Lakes, New Jersey, USA) until the centrifugation was stored on ice, spleen, and kidneys were removed, and subsequently, their wet weights were measured. After weighing, the kidneys were cut longitudinally, resulting in two half kidneys. One-half parts were stored in 10% buffered (pH 7,4) formalin solution for histopathological analysis. From the other half parts of the kidneys, the medulla and cortex were dissected and immediately freeze in dry ice. The prepared samples were stored at −80 °C.

### 4.5. Determination of Lipid Peroxidation and Antioxidant Parameters

Before the biochemical analyses, frozen kidney samples were completely homogenized by IKA Ultra Turrax instrument (IKA, Staufen, Germany) in 9-fold ice-cold (4 °C) physiological saline (0.85% *w/v* NaCl). The amount of conjugated dienes (CD), and in addition, conjugated trienes (CT) in the renal cortex samples, as specific markers of the lipid peroxidation at the initial phase, were measured at 232 and 268 nm after 2,2,4-trimethylpentane extraction [49]. Thiobarbituric reactive substances concentrations, as a marker of the lipid peroxidation at the terminal stage, were measured according to the method of Botsoglou et al. (1994) in native kidney homogenates and expressed as malondialdehyde, which served as standard [50].

In 10,000 g supernatant section of kidney homogenates, glutathione-peroxidase (GPx) activity was determined according to Lawrence and Burk [51]. This method was applied cumene-hydroperoxide as a substrate. The measurement of glutathione reductase (GR) activity was carried out according to Smith et al. (1988) [52], while in the case of glutathione-S-transferase (GST), the analytical procedure of Mannervik et al. [53] was used. Reduced glutathione (GSH) content and the concentration of glutathione disulfide (GSSG) were measured by Ellmann’s reagent according to the analytical procedure of Rahman et al. [54]. GSH and GSSG content and enzyme activities were calculated to the total protein content of the 10,000 g supernatant section of the renal cortex homogenate, which was determined by Folin-phenol reagent (Sigma- Aldrich, USA) according to the analytical method of Lowry et al. [55].

### 4.6. Quantitative Real-Time PCR

The renal cortex samples were completely homogenized by IKA Ultra Turrax T25 instrument in TRI Reagent Solution (Ambion, Austin, TX, United States). The total RNA was isolated from the samples using QIAGEN RNeasy Mini Kit (Qiagen, Hilden, Germany) according to the manufacturer’s instruction. The genomic DNA contaminations were eliminated by DN-ase on-column digestion. One hundred microliters RN-ase-free DNase I (1 unit DN-ase) (Thermo Scientific, San Jose, CA, USA) solution was applied in this reaction. Quality control and the quantitative measurements of the total RNA samples were performed with NanoDrop (Thermo Scientific, San Jose, CA, USA). The RT-minus controls did not show amplification. The cDNA synthesis was carried out using the High Capacity cDNA Reverse Transcription Kit (Applied Biosystems, Foster City, CA, USA). The oligonucleotide primers for the mRNA expression analysis (comparative Ct, qRT-PCR) were designed using the Primer Express 3.0 software. The oligonucleotides (Microsynth, Wien, Ausztria) were applied in the real-time PCR reactions. Fast EvaGreen qPCR Master Mix (Biotium, Hayward, CA, USA) served as a fluorescent dye and enzyme. The reactions were carried out on the ABI StepOnePlus instrument. The oligonucleotide primers were listed in Supplementary Material Table S1. The gene expression alterations were analyzed by ABI Step One 2.3 software (Thermo Scientific, San Jose, CA, USA). The integrities of the amplicons were tested with Melt Curve Analysis on the ABI Step OnePlus instrument. Expressions were normalized to mouse *ppia* (peptidylprolyl isomerase A) mRNA expression levels [56].

### 4.7. Western Hybridization

The renal cortex samples were completely homogenized by IKA Ultra Turrax T25 homogenizer in Radio Immunoprecipitation Assay (RIPA) solution. Phosphatase and protease inhibitor cocktail tablets were used to prevent the protein and the phosphate group degradation (Complete Mini and PhosSTOP, Roche, Basel, Switzerland) according to the manufacturer’s instruction. Total protein contents was determinate by the Pierce BCA Protein Assay Kit (Thermo Scientific, San Jose, CA, USA). Target proteins in the kidney tissue were measured by Western blot analysis using the following antibodies: rabbit antibodies against mouse NRF2 (bs-1074R) and NRF2 (Ser40) (bs-2013R) were purchased from Bioss (Bioss, Huissen, The Netherlands). Antibody against the B-actin housekeeping protein was purchased from Sigma (A5316 Ac74, Sigma, St. Louis, MO, USA). 

Samples containing 30 μg proteins were separated on 12% denaturating SDS-PAGE at 120 V for 2 h, and subsequently, the proteins were transferred to a Hybond-ECL membrane (Amersham Life Science, Amersham, UK) by using semi-dry transfer instrument (Trans Blot SD Cell, Biorad, Hercules, CA, USA). The transfer was performed at 24 V for 60 min. Cold Tris-Glycine-Methanol buffer (4 °C) was applied to this step. The membranes were incubated for 1 hour in blocking solution (1 × TBS, 0.05% Tween-20, 3% Cohn fraction V of BSA) and then incubated overnight in the same solution containing the given antibodies at 4 °C. The rabbit anti-NRF2 antibody was used at 1: 1000 dilutions, the rabbit anti–NRF2 (Ser40) antibody was used at 1:750 dilutions and the anti-b-actin antibody was used at 1:10,000 dilutions. The membranes were washed (three times) for 5 minutes in 1 × TBS, 0.05% Tween-20 buffer before a 2 h incubation in a solution (1 × TBS, 0.05% Tween-20 and 1% BSA Cohn fraction V) containing biotinylated anti-rabbit IgG (BA 1000, Vector, Burlingame, CA, USA) and anti-mouse IgG (BAA 2000, Vector, Burlingame, CA, USA) at 1:1000 dilution. The membranes were rinsed four times and developed by Vectastain Elite ABC Peroxidase Kit (Vector, Burlingame, CA, USA).

### 4.8. Histology

The kidney samples were fixed in formalin. We used the standard protocol for processing and paraffin embedding. The kidney sections (4 µm) were stained with eosin and hematoxylin. An expert veterinary pathologist analyzed the slides in a blind testing manner.

### 4.9. Statistical Analysis

Data are expressed and illustrated as means ± SD. The data were first analyzed by the Kolmogorov–Smirnov normality test. Passing this normality test, data were subjected to one-way ANOVA (followed by Tukey’s post hoc analysis). If the data showed non-Gaussian statistical distribution, the Kruskal–Wallis statistical method was applied to data analysis. GraphPad PRISM 6 software was applied to statistical analysis (GraphPad Software, 6.0, San Jose, CA, USA). *p* ≤ 0.05 was considered statistically significant.

## Figures and Tables

**Table 1:** Blood plasma ochratoxin-A (OTA) content in a single oral dose (24 h), repeated daily oral dose (72 h), and repeated daily oral dose (21 days) experiments.

Time	24 h	24 h	72 h	72 h	21 Days
Dose	single	single	repeated	repeated	repeated
	1 mg/kg bw	10 mg/kg bw	1 mg/kg bw	10 mg/kg bw	0.5 mg/kg bw
OTA level (ng/mL)	843.02 ± 285.16	2717.88 ± 391.52	269.73 ± 60.6	1969.28 ± 654.6	231.35 ± 50.23

## Supplementary Materials

The following are available online at https://www.mdpi.com/2072-6651/12/11/732/s1, Figure S1: Blood plasma OTA content in single oral dose (24 h), repeated daily oral dose (72 h) and repeated daily oral dose (21 days) experiments. A: OTA concentrations in blood plasma after single oral dose (24 h) mycotoxin administration at 1 mg/kg bw OTA (843.02 ± 285.16 ng/ml OTA in plasma) and 10 mg/kg bw OTA (2717.88 ± 391.52 ng/ml OTA in plasma) were applied. B: Repeated daily oral dose (72 h) mycotoxin treatment significantly elevated OTA concentration in the blood plasma in OTA 1 (269.73 ± 60.6 ng/ml OTA) and OTA 10 (1969.28 ± 654.6 ng/ml OTA) groups. C: Repeated daily oral dose (21 days) OTA treatment (0.5 mg/kg bw) resulted significantly elevated 231.35 ± 50.23 ng/ml OTA level in the blood plasma. Abbreviations: MMS–Group treated with methyl methanesulfonate, OTA 1 and OTA 10–Groups treated with 1 and 10 mg/kg bw ochratoxin A in single oral dose (24 h) and repeated daily oral dose (72 h) experiment, OTA 0.5–Group treated with 0.5 mg/kg bw ochratoxin A in repeated daily oral dose (21 days) experiment. Values are expressed as the mean ± S.D. Data were analysed by one-way ANOVA followed by the Tukey’s *post hoc* test. ** *p* < 0.01, *** *p* < 0.001, **** *p* < 0.0001 *vs.* vehicle. Figure S2: Effect of single oral dose (24 h) OTA administration on the spleen and kidney weight. A: The single oral OTA treatment (24 h) significantly elevated the normalized wet weight of the kidney in both OTA dose (*p* < 0.01 and 0.05). B: The normalized wet weights of spleen was significantly higher in the higher OTA treated group (* *p* < 0.05). Abbreviations: MMS–Group treated with methyl methanesulfonate, OTA 1 and OTA 10–Groups treated with 1 and 10 mg/kg bw ochratoxin A in acute experiment. Values are expressed as the mean ± S.D. Data were analysed by one-way ANOVA followed by the Tukey’s *post hoc* test. * *p* < 0.05, ** *p* < 0.01 *vs.* vehicle. Figure S3: Effect of repeated daily oral dose (72 h) OTA administration on the spleen and kidney weight. A: The repeated daily oral dose (72 h), 72 h, OTA toxicity did not influence significantly the wet weight of kidney. B: The normalized wet weights of spleen were significantly lower in both MMS and OTA treated groups, as compared to the control (** *p* < 0.01). Abbreviations: MMS–Group treated with methyl methanesulfonate, OTA 1 and OTA 10–Groups treated with 1 and 10 mg/kg bw ochratoxin A in repeated daily oral dose (72 h) experiment. Values are expressed as the mean ± S.D. Data were analysed by one-way ANOVA followed by the Tukey’s *post hoc* test. ** *p* < 0.01 *vs.* vehicle. Figure S4: Effect of repeated daily oral dose (21 days) OTA administration on the spleen and kidney weight. A: The repeated daily oral dose (21 days) OTA treatment decreased significantly the normalized wet weight of kidney in the OTA treated group. B: The normalized wet weight of spleen did not show statistically significant differences between the MMS and OTA treated groups (** *p* < 0.01). Abbreviations: MMS–Group treated with methyl methanesulfonate, OTA 0.5–Group treated with 0.5 mg/kg bw ochratoxin A in repeated daily oral dose (21 days) experiment. Values are expressed as the mean ± S.D. Data were analysed by one-way ANOVA followed by the Tukey’s *post hoc* test. ** *p* < 0.01 *vs.* vehicle. Figure S5: Histology of the kidneys following OTA treatment. Representative photomicrographs showing hematoxylin-eosine stained kidney sections. Abbreviations: (A) Vehicle, (B) OTA 0.5–Group treated with 0.5 mg/kg bw ochratoxin A in repeated daily oral dose (21 days) experiment. (C) OTA 1 and (D) OTA 10–Groups treated with 1 and 10 mg/kg bw ochratoxin A in repeated daily oral dose (72 h) experiment. Symbols: white arrow- detached necrotic epithelial cells, black arrow- necrotic tubular cells, white arrowhead- tubular cell regeneration. Scale bar: 100 µm. Figure S6: Effect of single oral dose (24 h) OTA expositions on the NRF2 Ser40-P protein expression levels in kidney. A: Quantification of protein expression normalized to vehicle treated controls by densitometry. Significant differences were not found in the OTA treated animals. B: Representative examples of Western blots using kidney homogenate from mice. Abbreviations: MMS–Group treated with methyl methanesulfonate, OTA 1 and OTA 10–Groups treated with 1 and 10 mg/kg bw ochratoxin A in single oral dose (24 h) experiment. Values are expressed as the mean ± S.D. Data were analysed by one-way ANOVA followed by the Tukey’s *post hoc* test. Figure S7: Effect of repeated daily oral dose (72 h) OTA expositions on the NRF2 Ser40-P protein expression levels in kidney. A: Quantification of protein expression normalized to vehicle treated controls by densitometry. Significant protein expression change was not found in the OTA treated animals. B: Representative examples of Western blots using kidney homogenate from mice. Abbreviations: MMS–Group treated with methyl methanesulfonate, OTA 1 and OTA 10–Groups treated with 1 and 10 mg/kg bw ochratoxin A in repeated daily oral dose (72 h) experiment. Values are expressed as the mean ± S.D. Data were analysed by one-way ANOVA followed by the Tukey’s *post hoc* test. Figure S8: Effect of repeated daily oral dose (21 days) OTA expositions on the NRF2 protein expression levels in kidney. A: Quantification of protein expression normalized to vehicle treated controls by densitometry. Significant protein expression elevation was found in the MMS treated animals, but the repeated daily oral dose (21 days) OTA administration did not influence the protein levels. B: Representative examples of Western blots using kidney homogenate from mice. Abbreviations: MMS–Group treated with methyl methanesulfonate, OTA 0.5–Group treated with 0.5 mg/kg bw ochratoxin A in repeated daily oral dose (21 days) experiment. Values are expressed as the mean ± S.D. Data were analysed by one-way ANOVA followed by the Tukey’s *post hoc* test. ** *p* < 0.01 *vs.* vehicle. Figure S9: Effect of repeated daily oral dose (21 days) OTA expositions on the NRF2 Ser40-P protein expression levels in kidney. A: Quantification of protein expression normalized to vehicle treated controls by densitometry. Significant protein expression elevation was found in the MMS treated animals, but the repeated daily oral dose (21 days) OTA administration did not influence the Ser40 phosphorylated NRF2 protein levels. B: Representative examples of Western blots using kidney homogenate from mice. Abbreviations: MMS–Group treated with methyl methanesulfonate, OTA 0.5–Group treated with 0.5 mg/kg bw Ochratoxin A in repeated daily oral dose (21 days) experiment. Values are expressed as the mean ± S.D. Data were analysed by one-way ANOVA followed by the Tukey’s *post hoc* test. * *p* < 0.05 *vs.* vehicle.
