# Supplementary material for: Effects of Single and Repeated Oral Doses of Ochratoxin A on the Lipid Peroxidation and Antioxidant Defense Systems in Mouse Kidneys
Source: Toxins (Basel). 2020 Nov 22;12(11):732. doi: 10.3390/toxins12110732 (PMC7700583; doi:10.3390/toxins12110732)
Supplement: Supplementary file 1 [file toxins-12-00732-s001.pdf]

Supplementary Materials: Effects of Single and Repeated Oral Doses of Ochratoxin A on the Lipid Peroxidation and Antioxidant Defense Systems in Mouse Kidneys

Szilamér Ferenczi, Dániel Kuti, Mátyás Cserháti, Csilla Krifaton, Sándor Szoboszlay, József Kukolya, Zsuzsanna Szőke, Mihály Albert, Balázs Kriszt, Krisztina J. Kovács, Miklós Mézes and Krisztián Balogh

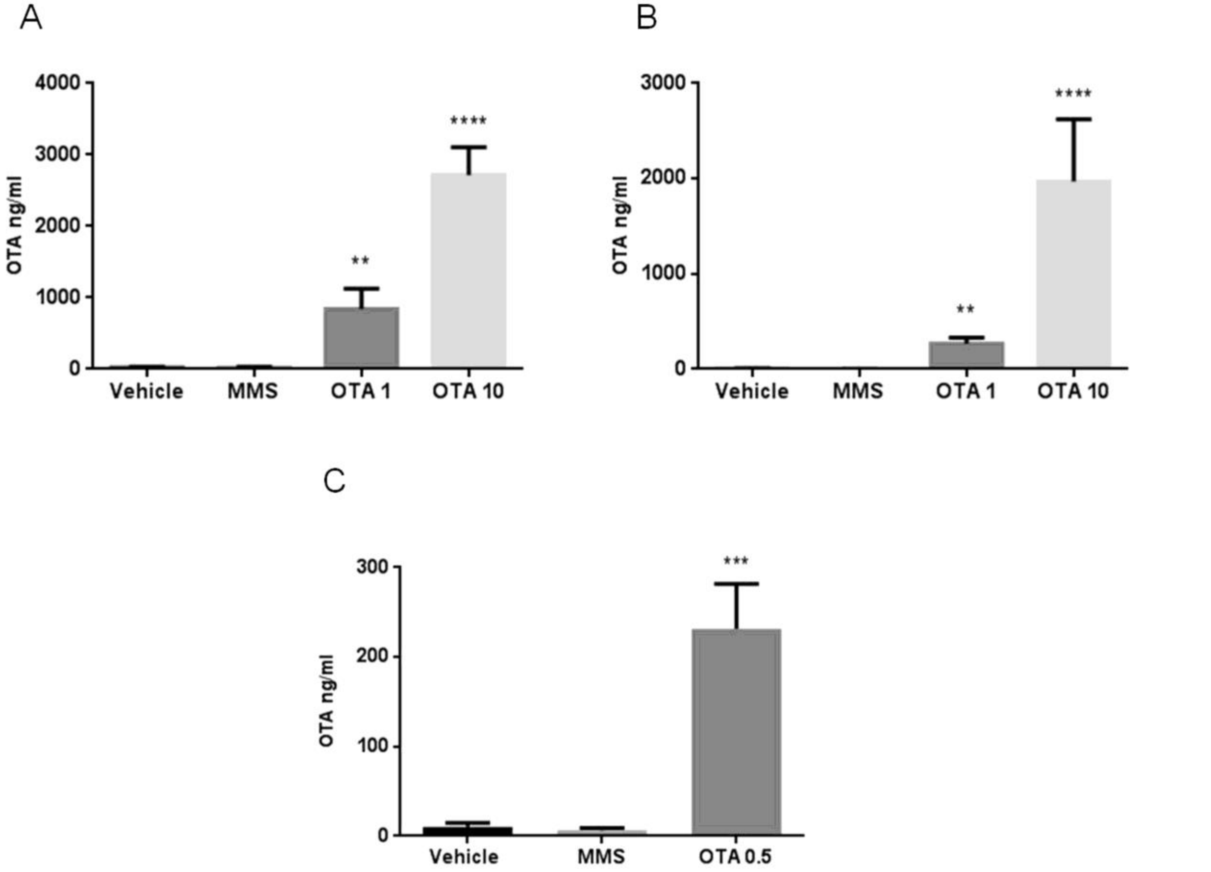

Figure S1. Blood plasma OTA content in single oral dose (24 hours), repeated daily oral dose (72 hours) and repeated daily oral dose (21 days) experiments. **A**: OTA concentrations in blood plasma after single oral dose (24 hours) mycotoxin administration at 1 mg/kg bw OTA (843.02 ± 285.16 ng/ml OTA in plasma) and 10 mg/kg bw OTA (2717.88 ± 391.52 ng/ml OTA in plasma) were applied. **B**: Repeated daily oral dose (72 hours) mycotoxin treatment significantly elevated OTA concentration in the blood plasma in OTA 1 (269.73±60.6 ng/ml OTA) and OTA 10 (1969.28 ± 654.6 ng/ml OTA) groups. **C**: Repeated daily oral dose (21 days) OTA treatment (0.5 mg/kg bw) resulted significantly elevated 231.35 ± 50.23 ng/ml OTA level in the blood plasma. Abbreviations: MMS – Group treated with methyl methanesulfonate, OTA 1 and OTA 10 – Groups treated with 1 and 10 mg/kg bw ochratoxin A in single oral dose (24 hours) and repeated daily oral dose (72 hours) experiment, OTA 0.5 – Group treated with 0.5 mg/kg bw ochratoxin A in repeated daily oral dose (21 days) experiment. Values are expressed as the mean± S.D. Data were analysed by one-way ANOVA followed by the Tukey's *post hoc* test.***p* < 0.01,****p* < 0.001, *****p* < 0.0001 *vs.* vehicle.

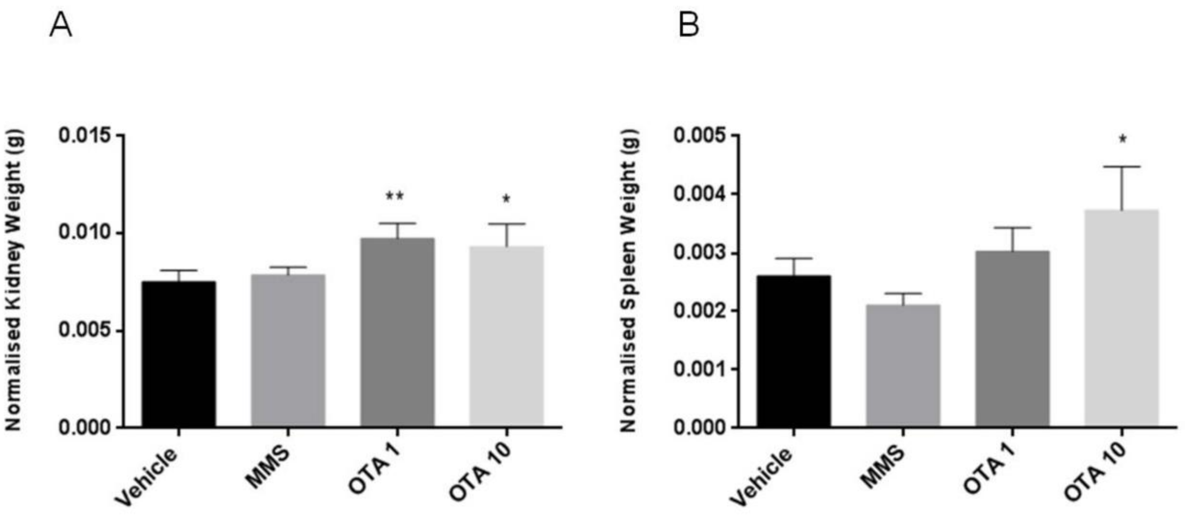

Figure S2. Effect of single oral dose (24 hours) OTA administration on the spleen and kidney weight. **A**: The single oral OTA treatment (24 hours) significantly elevated the normalized wet weight of the kidney in both OTA dose ($p < 0.01$ and 0.05). **B**: The normalized wet weights of spleen was significantly higher in the higher OTA treated group ($*p < 0.05$). Abbreviations: MMS – Group treated with methyl methanesulfonate, OTA 1 and OTA 10 – Groups treated with 1 and 10 mg/kg bw ochratoxin A in acute experiment. Values are expressed as the mean±S.D. Data were analysed by one-way ANOVA followed by the Tukey's *post hoc* test. $*p < 0.05$, $**p < 0.01$ *vs.* vehicle.

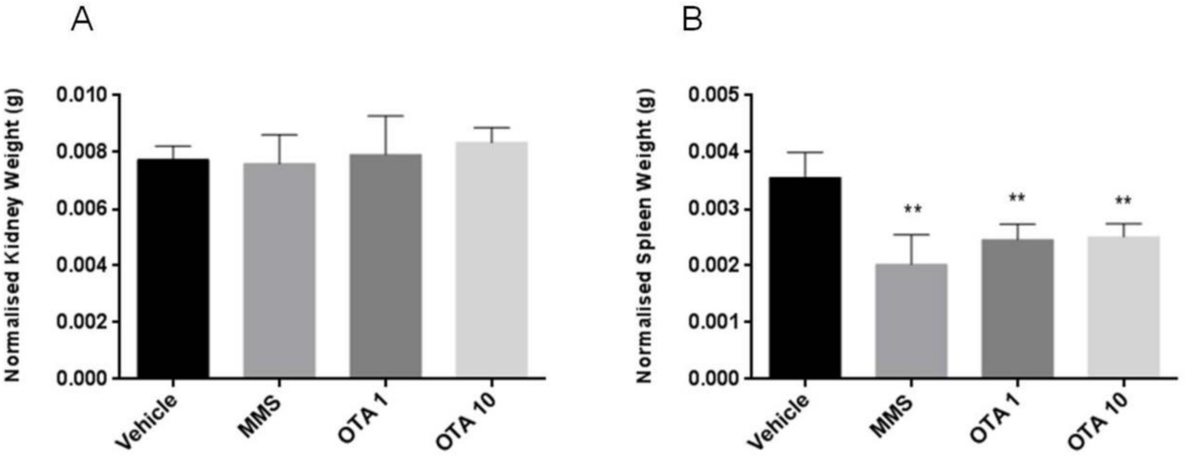

Figure S3. Effect of repeated daily oral dose (72 hours) OTA administration on the spleen and kidney weight. **A**: The repeated daily oral dose (72 hours), 72 hours, OTA toxicity did not influence significantly the wet weight of kidney. **B**: The normalized wet weights of spleen were significantly lower in both MMS and OTA treated groups, as compared to the control ($**p < 0.01$). Abbreviations: MMS – Group treated with methyl methanesulfonate, OTA 1 and OTA 10 – Groups treated with 1 and 10 mg/kg bw ochratoxin A in repeated daily oral dose (72 hours) experiment. Values are expressed as the mean±S.D. Data were analysed by one-way ANOVA followed by the Tukey's *post hoc* test. $**p < 0.01$ *vs.* vehicle.

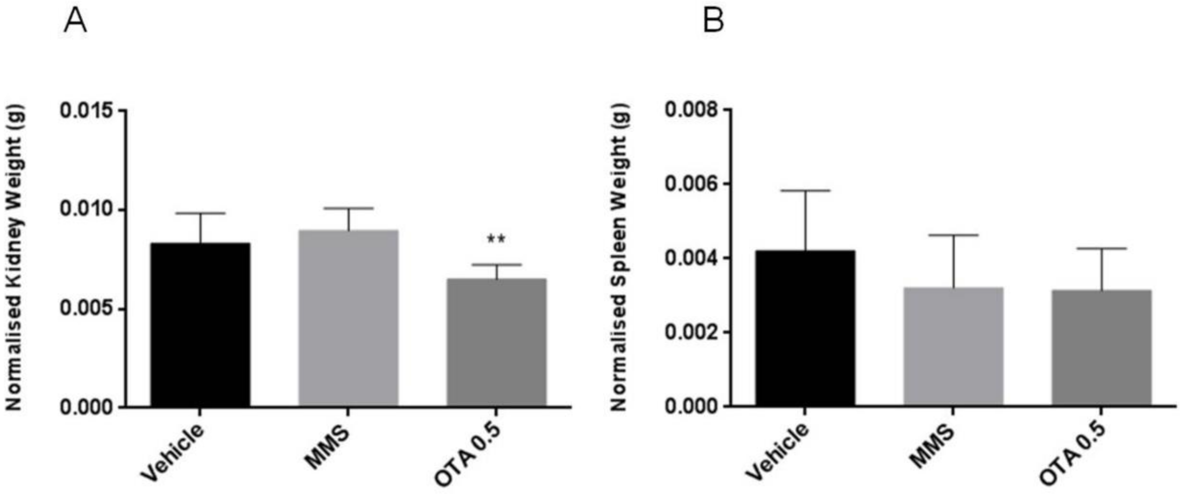

Figure S4. Effect of repeated daily oral dose (21 days) OTA administration on the spleen and kidney weight. **A**: The repeated daily oral dose (21 days) OTA treatment decreased significantly the normalized wet weight of kidney in the OTA treated group. **B**: The normalized wet weight of spleen did not show statistically significant differences between the MMS and OTA treated groups (**$p < 0.01$).Abbreviations: MMS – Group treated with methyl methanesulfonate, OTA 0.5 – Group treated with 0.5 mg/kg bw ochratoxin A in repeated daily oral dose (21 days) experiment. Values are expressed as the mean±S.D. Data were analysed by one-way ANOVA followed by the Tukey's *post hoc* test. **$p < 0.01$ *vs.* vehicle.

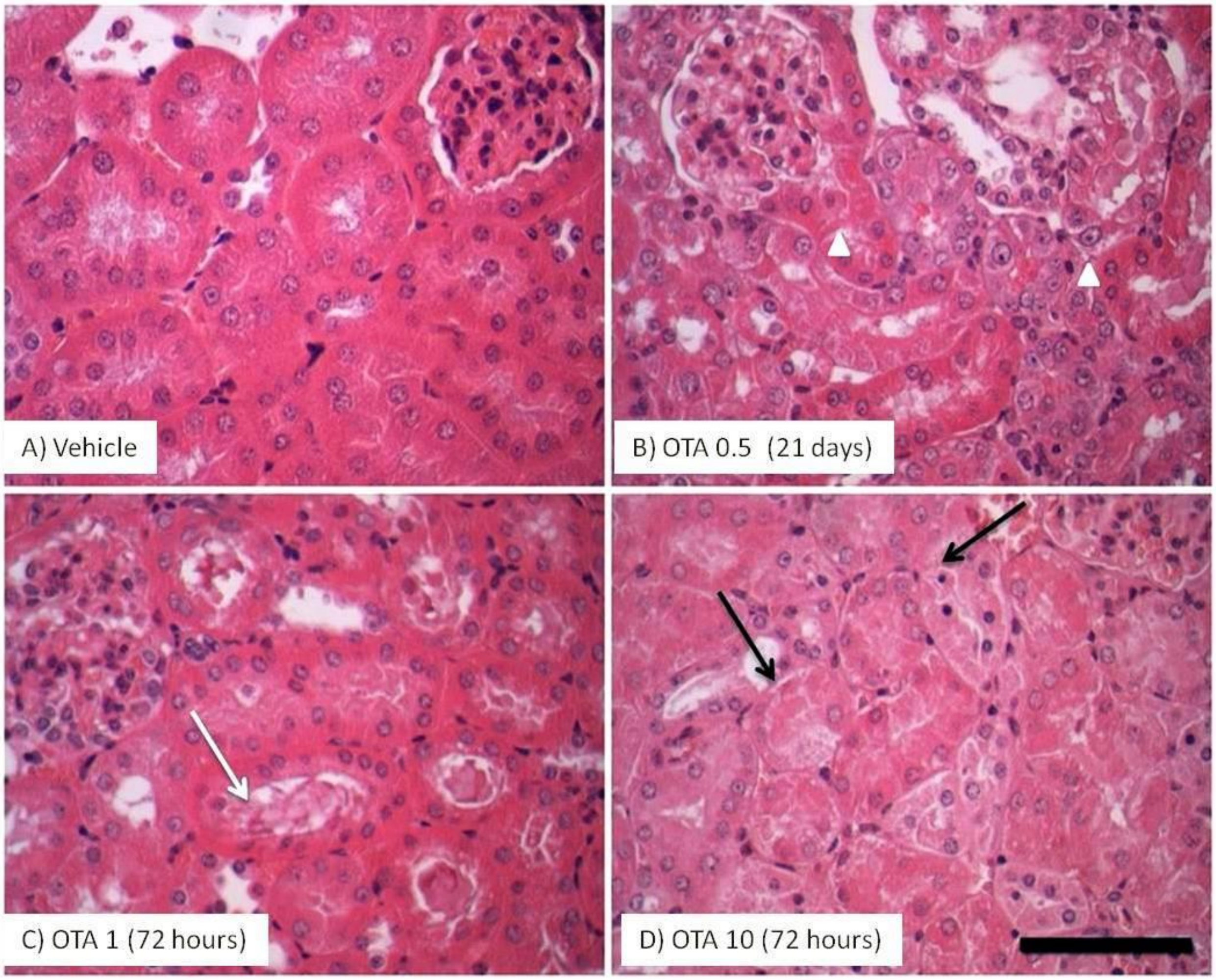

Figure S5. Histology of the kidneys following OTA treatment. Representative photomicrographs showing hematoxylin-eosine stained kidney sections. Abbreviations: (**A**) Vehicle, (**B**) OTA 0.5 – Group treated with 0.5 mg/kg bw ochratoxin A in repeated daily oral dose (21 days) experiment. (**C**) OTA 1 and (**D**) OTA 10 – Groups treated with 1 and 10 mg/kg bw ochratoxin A in repeated daily oral dose (72 hours) experiment. Symbols: white arrow- detached necrotic epithelial cells, black arrow- necrotic tubular cells, white arrowhead- tubular cell regeneration. Scale bar: 100 μm.

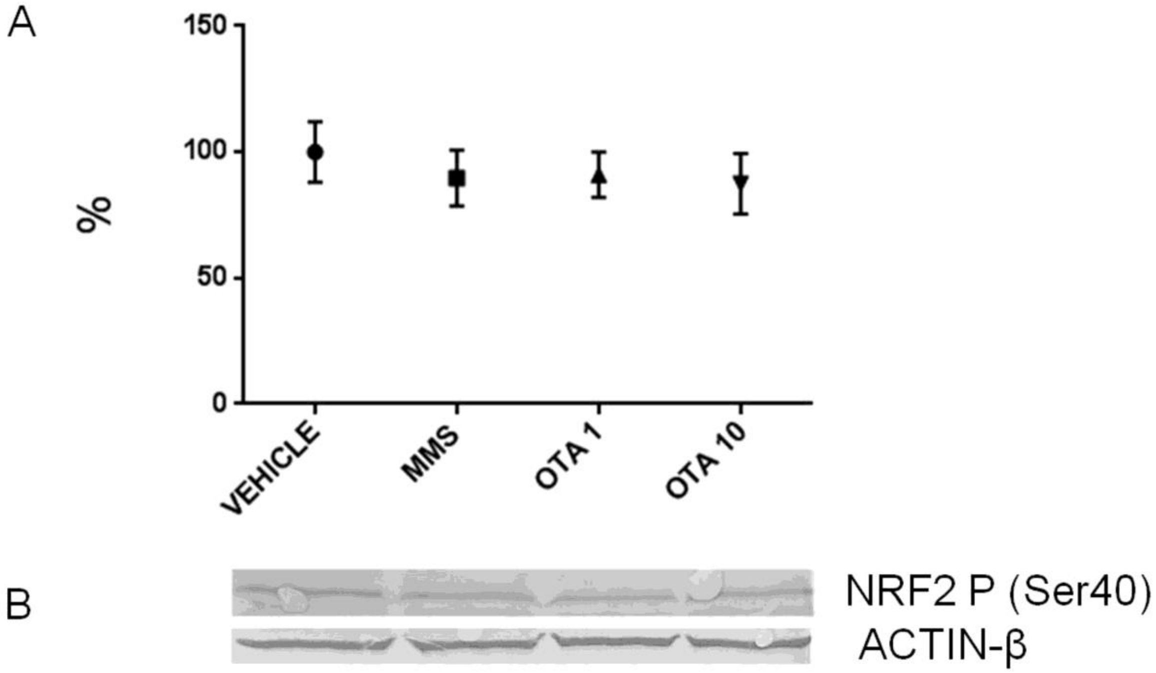

Figure S6. Effect of single oral dose (24 hours) OTA expositions on the NRF2 Ser40-P protein expression levels in kidney. **A**: Quantification of protein expression normalized to vehicle treated controls by densitometry. Significant differences were not found in the OTA treated animals. **B**: Representative examples of Western blots using kidney homogenate from mice. Abbreviations: MMS – Group treated with methyl methanesulfonate, OTA 1 and OTA 10 – Groups treated with 1 and 10 mg/kg bw ochratoxin A in single oral dose (24 hours) experiment. Values are expressed as the mean± S.D. Data were analysed by one-way ANOVA followed by the Tukey's *post hoc* test.

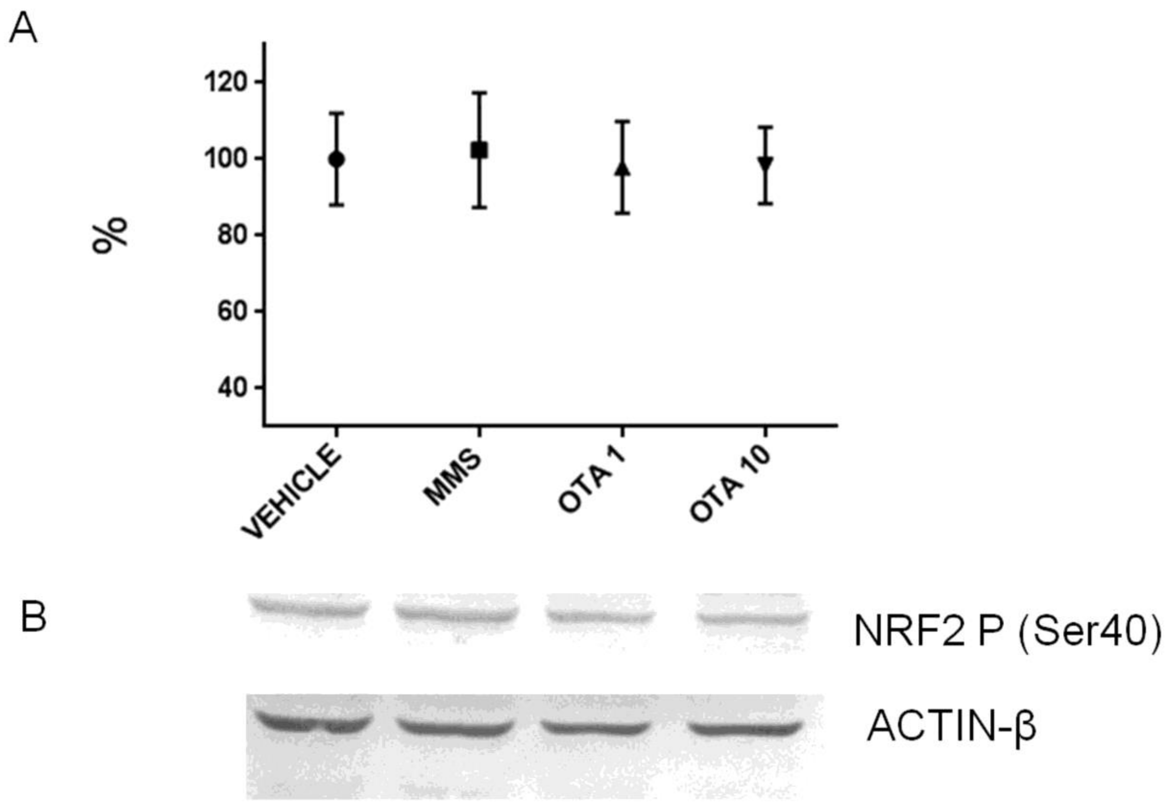

Figure S7. Effect of repeated daily oral dose (72 hours) OTA expositions on the NRF2 Ser40-P protein expression levels in kidney. **A**: Quantification of protein expression normalized to vehicle treated controls by densitometry. Significant protein expression change was not found in the OTA treated animals. **B**: Representative examples of Western blots using kidney homogenate from mice. Abbreviations: MMS – Group treated with methyl methanesulfonate, OTA 1 and OTA 10 – Groups treated with 1 and 10 mg/kg bw ochratoxin A in repeated daily oral dose (72 hours) experiment. Values are expressed as the mean± S.D. Data were analysed by one-way ANOVA followed by the Tukey's *post hoc* test.

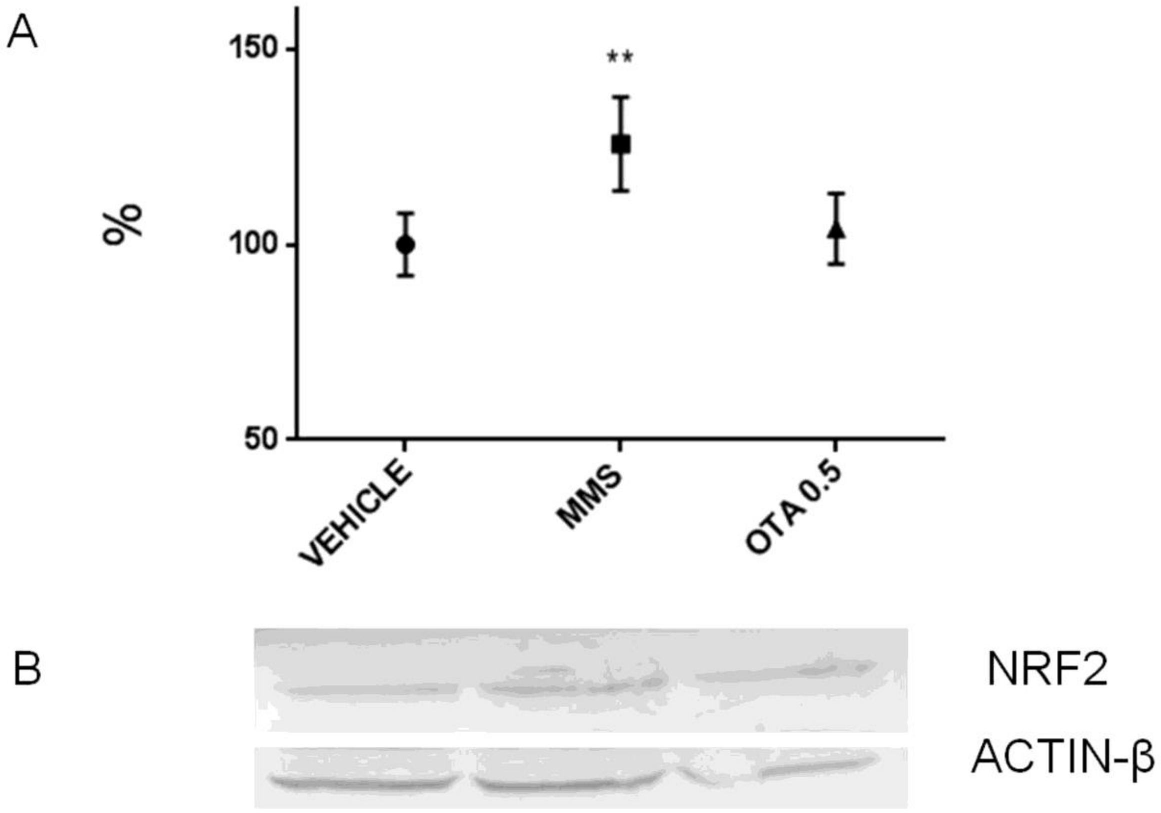

Figure S8. Effect of repeated daily oral dose (21 days) OTA expositions on the NRF2 protein expression levels in kidney. **A**: Quantification of protein expression normalized to vehicle treated controls by densitometry. Significant protein expression elevation was found in the MMS treated animals, but the repeated daily oral dose (21 days) OTA administration did not influence the protein levels. **B**: Representative examples of Western blots using kidney homogenate from mice. Abbreviations: MMS – Group treated with methyl methanesulfonate, OTA 0.5 – Group treated with 0.5 mg/kg bw ochratoxin A in repeated daily oral dose (21 days) experiment. Values are expressed as the mean± S.D. Data were analysed by one-way ANOVA followed by the Tukey's *post hoc* test.**$p < 0.01$ *vs.* vehicle.

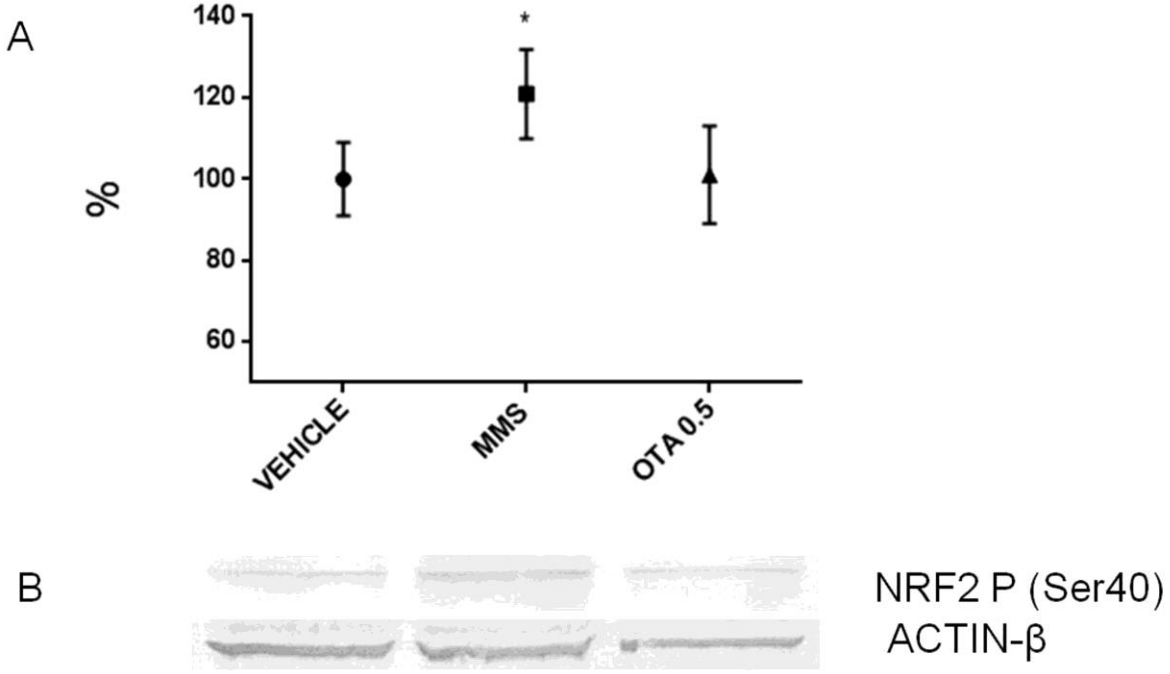

Figure S9. Effect of repeated daily oral dose (21 days) OTA expositions on the NRF2 Ser40-P protein expression levels in kidney. **A**: Quantification of protein expression normalized to vehicle treated controls by densitometry. Significant protein expression elevation was found in the MMS treated animals, but the repeated daily oral dose (21 days) OTA administration did not influence the Ser40 phosphorylated NRF2 protein levels. **B**: Representative examples of Western blots using kidney homogenate from mice. Abbreviations: MMS – Group treated with methyl methanesulfonate, OTA 0.5 – Group treated with 0.5 mg/kg bw Ochratoxin A in repeated daily oral dose (21 days) experiment. Values are expressed as the mean± S.D. Data were analysed by one-way ANOVA followed by the Tukey's *post hoc* test.*$p < 0.05$ *vs.* vehicle.

Table S1. The nucleotide sequence of the oligonucleotid primers.

Gene Names	Oligo Sequence
ppia f	CGCGTCTCCTTCGAGCTGTTTG
ppia r	TGTAAAGTCACCACCCTGGCACA
ho-1 f	GTCAAGCACAGGGTGACAGA
ho-1 r	GCAGTATCTTGCACCAGGCTA
sod1 f	CCGGCTTGGCATCCGTTATC
sod1 r	GGCACCACCGGGTTCAAATA
sod2 f	CAGCGGTCGTGTAAACTCAAT
sod2 r	CAGCGCGCCATAGTCGTAA
gsr f	TGGCACTTGCGTGAATGTTG
gsr r	TGTTCAGGCGGCTCACATAG
gpx1 f	GTTCGGACACCAGGAGAATGG
gpx1 r	GTCGGACGTACTTGAGGGAA
gss f	CTCCGACGTGGTGACGTATG
gss r	CAGAGCACTGGGTACTGGTG
nrf2 f	TCCTGCCAAACTTGCTCCAT
nrf2 r	ATCCCCAGCCACGCTGAAAG
nqo1 f	GGTAGCGGCTCCATGTACTC

nqo1 r	CGCAGGATGCCACTCTGAAT
gpx2 f	TCGGACATCAGGAGAACTGTC
gpx2 r	CCCCAGGTCGGACATACTTG
keap1 f	CTGAGCCAGCAACTCTGTGA
keap1 r	GATGCCTTCGATGGACACCA
rac1 f	CTACCCGCAGACAGACGTG
rac1 r	GGTAGGTGATGGGAGTCAGC
hace1 f	ACTGACTCGGCTTAGCATCC
hace 1 r	TCCATCGCTCTCTCCATCCT
gsta1 f	AAGCAAGGAAGGCTTTCAAGATTCA
gsta1 r	TGCAAACCATTAGAGGCCAGTATC
